# Dopaminergic drugs modulate fear extinction-related processes in humans, but effects are mild

**DOI:** 10.1093/braincomms/fcaf333

**Published:** 2025-09-08

**Authors:** Alice Doubliez, Kristina Köster, Lara Müntefering, Enzo Nio, Nicolas Diekmann, Andreas Thieme, Bilge Albayrak, Seyed Ali Nicksirat, Friedrich Erdlenbruch, Giorgi Batsikadze, Thomas Michael Ernst, Sen Cheng, Christian Josef Merz, Dagmar Timmann

**Affiliations:** Department of Neurology and Center for Translational Neuro- and Behavioral Sciences (C-TNBS), Essen University Hospital, University of Duisburg-Essen, 45147 Essen, Germany; Department of Neurology and Center for Translational Neuro- and Behavioral Sciences (C-TNBS), Essen University Hospital, University of Duisburg-Essen, 45147 Essen, Germany; Department of Neurology and Center for Translational Neuro- and Behavioral Sciences (C-TNBS), Essen University Hospital, University of Duisburg-Essen, 45147 Essen, Germany; Department of Neurology and Center for Translational Neuro- and Behavioral Sciences (C-TNBS), Essen University Hospital, University of Duisburg-Essen, 45147 Essen, Germany; Institute for Neural Computation, Faculty of Computer Science, Ruhr University Bochum, Bochum 44801, Germany; Department of Neurology and Center for Translational Neuro- and Behavioral Sciences (C-TNBS), Essen University Hospital, University of Duisburg-Essen, 45147 Essen, Germany; Department of Pediatrics I and C-TNBS, Essen University Hospital, University of Duisburg-Essen, Essen 45147, Germany; Department of Neurology and Center for Translational Neuro- and Behavioral Sciences (C-TNBS), Essen University Hospital, University of Duisburg-Essen, 45147 Essen, Germany; Department of Neurology and Center for Translational Neuro- and Behavioral Sciences (C-TNBS), Essen University Hospital, University of Duisburg-Essen, 45147 Essen, Germany; Department of Neurology and Center for Translational Neuro- and Behavioral Sciences (C-TNBS), Essen University Hospital, University of Duisburg-Essen, 45147 Essen, Germany; Department of Neurology and Center for Translational Neuro- and Behavioral Sciences (C-TNBS), Essen University Hospital, University of Duisburg-Essen, 45147 Essen, Germany; Institute for Neural Computation, Faculty of Computer Science, Ruhr University Bochum, Bochum 44801, Germany; Department of Cognitive Psychology, Institute of Cognitive Neuroscience, Ruhr University Bochum, Bochum 44780, Germany; Department of Neurology and Center for Translational Neuro- and Behavioral Sciences (C-TNBS), Essen University Hospital, University of Duisburg-Essen, 45147 Essen, Germany

**Keywords:** fear conditioning, dopamine, recall, memory consolidation, delay conditioning

## Abstract

The ability to extinguish learned fear responses is crucial for adaptive behaviour. The mesolimbic dopaminergic system originating in the ventral tegmental area has been proposed to contribute to fear extinction learning because of its critical role in reward learning. The unexpected omission of aversive unconditioned stimuli (US) is considered rewarding (outcome better than expected) and drives extinction learning. We tested the hypothesis that extinction learning is facilitated by dopaminergic drugs and impeded by anti-dopaminergic drugs. The effects of dopamine agonists [levodopa (100 mg) and bromocriptine (1.25 mg)] and antagonists [tiapride (100 mg) and haloperidol (3 mg)] on fear extinction learning were compared with placebo in 146 young and healthy human participants. A 3-day differential fear-conditioning paradigm was performed with pupil size and skin conductance responses (SCRs) being recorded. Participants underwent fear acquisition training on Day 1, extinction training on Day 2 and recall on Day 3. The conditioned stimuli (CS+, CS−) consisted of two geometric figures. A short electrical stimulation was used as the aversive US. One of the four drugs or placebo was administered prior to the extinction phase on Day 2. Overall, effects were small and seen only in the bromocriptine group. In accordance with our hypothesis, we measured reduced pupil dilation during late recall in the bromocriptine group compared with the placebo group, indicating faster re-extinction of spontaneously recovered fear reactions on the third day. The effects of levodopa and haloperidol were unspecific and related to generally increased SCR levels in the levodopa group (already prior to drug intake) and miotic side-effects of haloperidol. These findings provide additional support that the dopaminergic system contributes to extinction learning in humans, possibly by improving consolidation of fear extinction memory.

## Teaser

Doubliez *et al*. report dopaminergic and anti-dopaminergic drug effects on fear extinction learning and recall. They found that bromocriptine facilitated re-extinction of spontaneously recovered fear responses, evidenced by reduced pupil dilation during late recall. Other drugs showed unspecific effects. While dopaminergic drugs show potential in modulating fear extinction and recall, their effects are small.

## Graphical Abstract

**Graphical Abstract:**
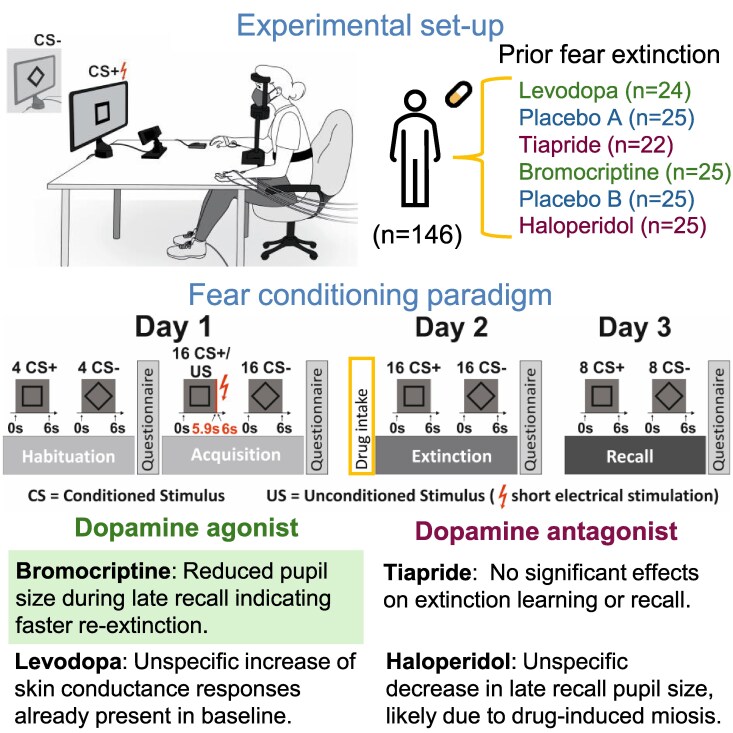

## Introduction

Learning to identify and react to threatening situations is essential for survival, but it is equally vital to adjust the behavioural responses when those stimuli are no longer associated with danger.^1^ The inability to extinguish fearful memories is an inherent aspect of many anxiety disorders, such as post-traumatic stress disorder and phobias.^2,3^ A widely used experimental approach to study learned fear responses and their subsequent extinction is based on Pavlovian conditioning.^4^ Fear conditioning assesses defensive responses elicited not only by an innate adverse stimulus but also by a conditioned stimulus (CS+) that was previously neutral and comes to predict the adverse event. During fear acquisition training, a neutral CS, when consistently paired with an aversive unconditioned stimulus (US), comes to evoke fear responses upon its onset. Fear extinction occurs when the CS+ is repeatedly presented in the absence of the previously paired US, leading to a gradual decrease in learned fear conditioned responses (CRs). Fear conditioning can be studied in both animals and humans. Behavioural responses, such as freezing, are commonly assessed to measure fear learning and extinction learning in rodents. However, the strength of the US in human fear-conditioning paradigms is rarely of sufficient magnitude to generate such defensive behaviours. Instead, physiological indicators like skin conductance responses (SCRs), heart rate and pupillary responses, as well as subjective responses based on questionnaires are employed to assess fear-related learning.^5^

Classical theories of associative learning propose that new learning arises from the discrepancy between predicted and actual outcomes, corresponding to a prediction error (PE).^6^ It is well established that the mesolimbic dopaminergic pathways play a critical role in reward processing and reinforcement learning. The release of dopamine (DA), originating from the ventral tegmental area (VTA), in the nucleus accumbens (NAc) is a phenomenon that has been consistently observed towards unexpected rewarding stimuli in associative learning tasks^7-9^. In extinction learning, omission of an expected aversive US constitutes a better-than-expected outcome, which may be processed as a reward PE^10-12^. Following this reasoning, it has been proposed that reward PE drive fear extinction learning and are also mediated by DA release from neurons originating in the VTA.^10^ In support of this hypothesis, recent studies conducted in rodents reported activation of a subset of DA neurons in the VTA following unexpected US omission during fear extinction,^13-16^ especially during early trials when the PE is the highest.^13,15^ Moreover, optogenetically inhibiting or activating those DA neurons at the time of US omission is sufficient to impair or enhance, respectively, fear extinction learning.^13^ Evidence suggests that the VTA DA neurons involved in fear extinction project and release DA in the NAc.^17^ Further supporting this observation, direct administration of a D2 receptor antagonist, haloperidol, into NAc of rats was found to impair fear extinction.^18^ This mechanism may be evolutionarily conserved, as similar dopaminergic responses to US omission have also been reported in invertebrates like Drosophila.^19,20^

Consistently, VTA activation has recently been observed in human fMRI during unexpected US omission in early fear extinction.^21^ To further understand the influence of DA on emotional learning processes in humans, dopaminergic pharmacological approaches during associative learning tasks have been employed.^22-25^ These interventions typically involve the intake of DA agonists or antagonists that predominantly interact with either D1 or D2 receptors, thereby either enhancing dopaminergic effects (agonists) or inhibiting them (antagonists). Lissek and colleagues evaluated the systemic effect of the DA antagonist tiapride and DA agonist bromocriptine during a cognitive predictive learning task. Drugs were administered prior to extinction. Whereas tiapride intake impaired extinction learning when occurring in a novel context,^23^ bromocriptine administration resulted in a significantly higher level of renewal in participants exhibiting renewal effects^24^ (i.e. return of the extinguished association in the acquisition context). These findings suggest that inhibiting DA leads to difficulties in the extinction of previously learned associations when the context changes, while increasing DA levels enhances context-related processes of learned associations. Moreover, in a fear-conditioning paradigm, administration of levodopa following fear extinction training and thus targeting extinction memory consolidation resulted in an enhancement of extinction memories during recall in humans.^22,25^ Findings appeared to depend on the success of fear extinction learning.^26^

To further investigate the effect of the dopaminergic system on fear extinction learning, we conducted a 3-day fear-conditioning study in healthy human participants who received a systemic single dose of either a DA agonist, a DA antagonist or a placebo prior to fear extinction training. Consistent with the known dopaminergic signals in reward-associated learning, we hypothesized that DA agonists enhance fear extinction learning and thus reduce recall of learned fear responses, whereas DA antagonists impair fear extinction learning and increase recall of learned fear responses following extinction learning.

## Materials and methods

### Participants

In total, 160 young and healthy participants (18–35 years) were recruited from university campuses and through online/offline advertisements. They received monetary compensation for their participation. Four participants were excluded due to technical issues, five for incomplete participation, one for lacking contingency awareness and four based on elevated scores on the Depression Anxiety Stress Scale (DASS-21-G).^27^ A total of 146 participants (74 women and 72 men, *Table 1*) were included in the analysis. Participants were fluent in German, did not smoke or use substances affecting the central nervous system (medication or illicit drugs), had no personal or family history of psychiatric or neurological disorders, had no contraindications to dopaminergic/anti-dopaminergic drugs, and had never taken part in similar learning experiments. A physician evaluation and electrocardiogram, including QTc interval assessment, were conducted to rule out cardiac contraindications. Additionally, female participants were included only if they were neither pregnant nor breastfeeding and were not using hormonal contraceptives. Participants scoring above moderate thresholds on the DASS-21-G scale (>20 depression, >14 anxiety and >25 stress) were excluded. The study was approved by the University Hospital Essen ethics committee and conducted in accordance with the Declaration of Helsinki. Informed consent was obtained from all participants by a physician before the experiment.

**Table 1:** Demographic characteristics and Depression Anxiety Stress Scale Scores (DASS-21-G) across medication groups, presented as mean (± standard deviation) unless otherwise specified; education reflects total years in the education system, and handedness (Edinburgh Handedness Inventory) classifies participants as right-, left-, or ambidextrous (ambidext.).

	Group A			Group B		
	Levodopa	Placebo A	Tiapride	Bromocriptine	Placebo B	Haloperidol
**Demographic**						
Participants (***n***)	24	25	22	25	25	25
Female (%)	54.2%	56%	50%	48%	44%	52%
Age (years)	24.54 ± 3.99	24.68 ± 4.36	23.68 ± 3.99	24.16 ± 3.42	24.92 ± 4.51	25.00 ± 3.65
Education level (years)	15.42 ± 2.04	16.94 ± 3.62	15.70 ± 1.86	15.40 ± 2.67	16.74 ± 3.12	16.34 ± 3.10
Edinburgh Handedness Inventory (***n***)	Right: 21 Left: 2 Ambidext.: 1	Right: 22 Left: 2 Ambidext.: 1	Right: 18 Left: 4 Ambidext.: 0	Right: 23 Left: 1 Ambidext.: 1	Right: 23 Left: 1 Ambidext.: 1	Right: 21 Left: 4 Ambidext.: 0
**Depression Anxiety Stress Scale**						
Depression	3.54 ± 2.25	2.6 ± 1.76	3.09 ± 2.35	3.40 ± 2.66	3.16 ± 2.53	3.04 ± 2.46
Anxiety	2.92 ± 1.91	2.16 ± 1.37	2.77 ± 1.66	2.04 ± 1.06	2.20 ± 1.47	2.16 ± 1.68
Stress	4.46 ± 2.93	4.04 ± 2.26	4.00 ± 3.02	4 ± 2.60	3.96 ± 2.70	4.64 ± 3.15

### Study design

**Figure 1:**
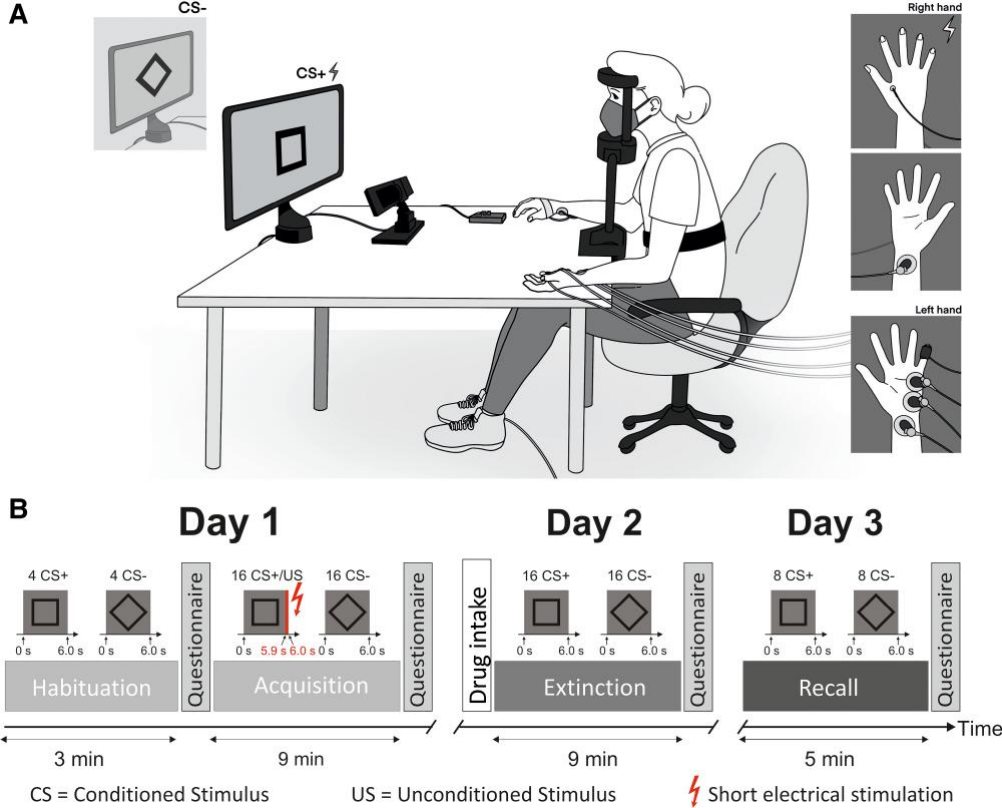
**Experimental setup and 3-day fear-conditioning paradigm.** (**A**) Illustration of the experimental setup representing participants sitting in front of a computer screen displaying black-and-white geometric figure as CS, while their pupil size and SCR were recorded. Note that participants wore face masks because the experiment was performed during the COVID-19 pandemic. (**B**) Three-day fear-conditioning paradigm. On Day 1, participants underwent habituation and acquisition training, during which CS+ trials were paired with a short electrical stimulation US. Day 2 begins with drug intake and is followed by extinction training. On Day 3, the recall phase takes place.

Pupil size and SCRs were monitored while participants completed a 3-day differential fear-conditioning paradigm (Fig. 1A). As the times to peak concentration differed between the four drugs, participants were randomly assigned to two groups (A and B; *Table 1*). Group A participants received either placebo, levodopa or tiapride, while Group B participants received either placebo, bromocriptine or haloperidol. Participants were randomly assigned to one of three drug groups, with randomization ensuring balanced sex distribution. Experiments were conducted under low lighting conditions for Group A and ambient lighting for Group B. The lighting difference was motivated by pupil measurement considerations: while low-light conditions were initially used, ambient lighting helps prevent pre-dilation of the pupil and reduces visual contrast with the screen. A placebo group was included in both lighting conditions, allowing within-condition comparisons.

Pupil size was monitored using an EyeLink® 1000 Plus eye tracking system (SR Research Ltd, Ontario, Canada), positioned 60 cm away from the participants. Participants used a headrest to maintain alignment with the recording camera and the display screen. Additionally, the eye-tracking system was calibrated prior to each phase. Skin conductance was recorded through an MP160 Data Acquisition Hardware unit (BIOPAC Systems Inc., Goleta, CA), with two electrodes affixed to the participant's left-hand hypothenar eminence (Fig. 1A).

A brief electrical stimulation served as the aversive US and was delivered to the first dorsal interosseous muscle of the right hand using a DS7A constant current stimulator (Digitimer Ltd, London, UK) via a 6.5-mm WASP concentric surface electrode (Specialty Developments, Bexley, UK). The electrode position was marked on Day 1 for consistent positioning on the following days. The 100 ms US consisted of four consecutive 500 µs pulses, spaced 33 ms apart, with a maximum stimulation voltage of 400 V. Stimulation intensity was adjusted individually to be highly uncomfortable but not painful. US intensity was increased by 20%, the same as in Inoue *et al*.,^28^ and remained constant across all days. The electrical stimulation electrode was attached throughout the experiment, providing stimulation only during paired CS+/US trials. Following local COVID-19 rules, participants and investigators wore face masks. This study was preregistered on Open Science Framework (https://doi.org/10.17605/OSF.IO/A3KJN).

The sample size was determined separately for Groups A and B using G*Power 3.1.9.7 for repeated measures analyses of variance (ANOVAs) within-between interactions. Our goal was to obtain 0.85 power (1 − *β*) to detect a medium effect size *f* = 0.25^29,30^ at the standard 0.05 alpha error probability and an assumed correlation among repeated measurements of *r* = 0.20.^31^ The analysis indicated a required sample size of 75 participants per group, which was achieved for Group B but fell short by 4 participants in Group A.

### Fear-conditioning paradigm

This 3-day differential fear-conditioning paradigm was adapted from Ernst *et al*.^32^ Participants were asked to remain still and focus on the screen displaying the paradigm using Presentation software (version 22.1, Neurobehavioral System Inc., Berkeley, CA). Two pictures of black-and-grey geometric figures, a square and a diamond (square titled by 45°), were used as visual conditioned stimuli (either CS+ or CS−) (Fig. 1A and B). The visual CS paired with the electrical US was randomly assigned and remained the same throughout the experiment.

During the experiment, participants encountered three types of trials: CS+ co-terminating with a US (paired CS+/US trial), CS+ without a US (CS + only trial) and CS− that was never followed by a US. For CS+/US paired trials, the US was administered 5.9 s after CS+ onset. Inter-trial intervals featured a black cross on a grey background and varied from 6 to 12 s. The paradigm included a habituation phase (4 CS+ trials and 4 CS− trials, in alternating order) followed by fear acquisition training (16 paired CS+/US trials—100% reinforcement rate and 16 CS− trials) on Day 1, extinction training (16 CS+ trials and 16 CS− trials) on Day 2 and a recall test (8 CS+ and 8 CS−) on Day 3 (Fig. 1B). A volatile phase followed the recall test on Day 3, as preregistered, but was not analysed as it was unrelated to our research questions. Full reinforcement rate was used during fear acquisition training to facilitate maximal PE during the early phase of extinction training. The order of trial types presented within each phase was pseudo-randomized, following the approach used by Ernst *et al*.^32^

### Drug treatment

Prior to extinction training on Day 2, a single dose of levodopa/carbidopa (100/25 mg; referred to as ‘levodopa’ hereafter),^33^ bromocriptine (1.25 mg),^24,34-37^ tiapride (100 mg),^23^ haloperidol (3 mg) or placebo was given to participants in non-transparent capsules. Drugs were administered at a specific time prior to extinction training, assuring maximum serum levels at the beginning of extinction training (60 min for levodopa^38^; 90 min for bromocriptine^39^; 120 min for haloperidol^40^ and tiapride^41^). Prior to levodopa or bromocriptine intake, 20 mg domperidone^42^ was given to prevent nausea. To ensure a double-blind experiment, a placebo capsule was ingested at a corresponding time after tiapride or haloperidol intake, matching the total number of capsules ingested (Table S1). Blinding was maintained for all involved, from enrolment to data analysis completion. Participants fasted for 2 h before drug intake, and blood samples were collected daily after the experiment for external drug concentration analysis (Table S2).

### Questionnaires and contingency awareness

Participants were informed that electrical stimuli (US) might occur, and that the CS and US presentation patterns would remain consistent throughout the experiment. However, they remained unaware of the specific CS/US contingencies or whether and when the US would be presented.

To evaluate contingency awareness, participants specified which of the two CSs had been followed by a US at the end of each phase where they reported receiving electric stimuli. Moreover, participants had to indicate whether a US was expected after the CS presentation and, if so, to estimate the number and percentage of US that occurred after the respective CS presentation (US expectancy) during that phase. Regardless of US perception, participants used nine-step Likert scales to rate both CS respective valence, arousal, fear, US expectancy and US unpleasantness at the end of all phases.^43^

### SCR analysis

Skin conductance signals were recorded using an EDA 100C-MRI system (BIOPAC Systems Inc., Goleta, CA), which applied a 10 Hz low-pass filter to suppress high-frequency noise. Using semi-automated peak detection in MATLAB, SCRs were defined as the maximum trough-to-peak amplitude detected within a specified time window following CS onset.^44^ Detection thresholds were set to a minimum amplitude of 0.01 μS and a minimum rise time of 500 ms.^45^ Trials that did not meet the criteria were treated as non-responses (scored as zero) and retained for analysis. SCRs were evaluated within a time window from 1.0 to 5.9 s following CS onset. To reduce inter-individual variability, the resulting raw SCR amplitudes were baseline-shifted by adding 1 μS and subsequently normalized via a logarithmic transformation (LN(1 + SCR)).^45,46^

### Pupillometry analysis

Raw pupil data were preprocessed in MATLAB (version 9.13 (R2022b), MathWorks, Natick, USA) following the method proposed by Kret and Sjak-Shie^47^ guidelines method. Only trials where eyes were opened and looking at the visual stimuli (CS) in the centre of the screen were retained. Invalid samples were identified following multiple steps: (i) dilatation-speed outliers and edge artefacts, which compared changes in pupil width relative to adjacent samples; (ii) trendline-deviation outliers, which assessed the ratio of pupil area to adjacent samples; and (iii) temporally isolated samples outliers, underlying single data points separated from adjacent samples. Missing data points were interpolated for gaps shorter than 250 ms. Trials with major artefacts or many blinks were excluded from the analyses. Furthermore, a visual inspection of both raw and processed pupil data was performed to evaluate signal quality and decide whether recording from one or both eyes should be included in the rest of the analysis. If one eye showed significantly more artefacts, it was omitted from the analysis. Analysis focused on the 2 s preceding US onset, which correspond to the time interval that measures the largest difference between CS+ and CS− during fear acquisition training.^31^ Baseline correction was applied by subtracting the average pupil size during the 300 ms preceding CS onset from the corresponding pupil during CS presentation.^31,48^

### Statistical analysis

Primary outcome measures included SCRs and pupil size. For each phase (fear acquisition training, extinction training and recall test) CS+ and CS− were grouped into early and late blocks of four trials, each corresponding to the first and second half of the phase (i.e. the first 4 CS+ trials of fear acquisition training constituted the early acquisition block, and the last 4 CS+ trials correspond to late acquisition block). SCRs and pupil size were analysed separately using non-parametric repeated-measures ANOVA-type statistics (ATS) via the PROC Mixed procedure in SAS (SAS Studio 3.8, SAS Institute Inc, Cary, NC, USA). These analyses included medication subgroup as a between-subjects factor and stimulus type (CS+ and CS−) and block (early and late phase) as within-subjects factors. Additionally, a separate non-parametric ATS analysis was conducted on the first trial of the recall phase, with medication subgroup as a between-subjects factor and stimulus type (CS+ and CS−) as a within-subjects factor.

Self-reported ratings were evaluated using non-parametric ATS for repeated measures. In the analysis, phase (post-habituation, post-acquisition, post-extinction and post-recall) and stimulus type (CS+ versus CS−) were treated as within-subject factors, medication group as a between-subjects factor and rating type (arousal, valence, fear or US expectancy) as the dependent variable.

To assess the effect of drug intake on baseline pupil size, we conducted a non-parametric ATS within each medication group to compare baseline pupil size across all experimental phases. Significant results from the non-parametric ATS were followed by post-hoc comparisons using least square means tests. Dunnett's adjustment was applied to control for multiple comparisons when comparing each treatment group to their respective control conditions. Tukey's test was used when comparing other conditions within each medication group.

Additional exploratory analyses, including correlations with serum drug concentrations and baseline psychological traits, as well as subgroup analyses based on extinction success, are reported in the Supplementary Materials.

## Results group A (levodopa, placebo and tiapride)

### Questionnaires

Across all participants, CS+ was rated significantly higher in unpleasantness (valence), arousal and fear compared with CS− after fear acquisition training. Although this difference remained statistically significant during extinction and recall, CS+ ratings decreased over time, reflecting successful extinction learning (Fig. S1; Table S3; Table S4). Participants estimated a high likelihood of US occurrence following CS+ (98.21 ± 11.40%), while the probability was minimal for CS− (1.54 ± 5.34%; Table S5). Valence, fear, arousal and US expectancy ratings are detailed in the supplement, along with information on CS–US contingency awareness and US unpleasantness (Fig. S1; Table S3; Table S4; Table S5).

### Pupillometry

#### Habituation phase

**Figure 2:**
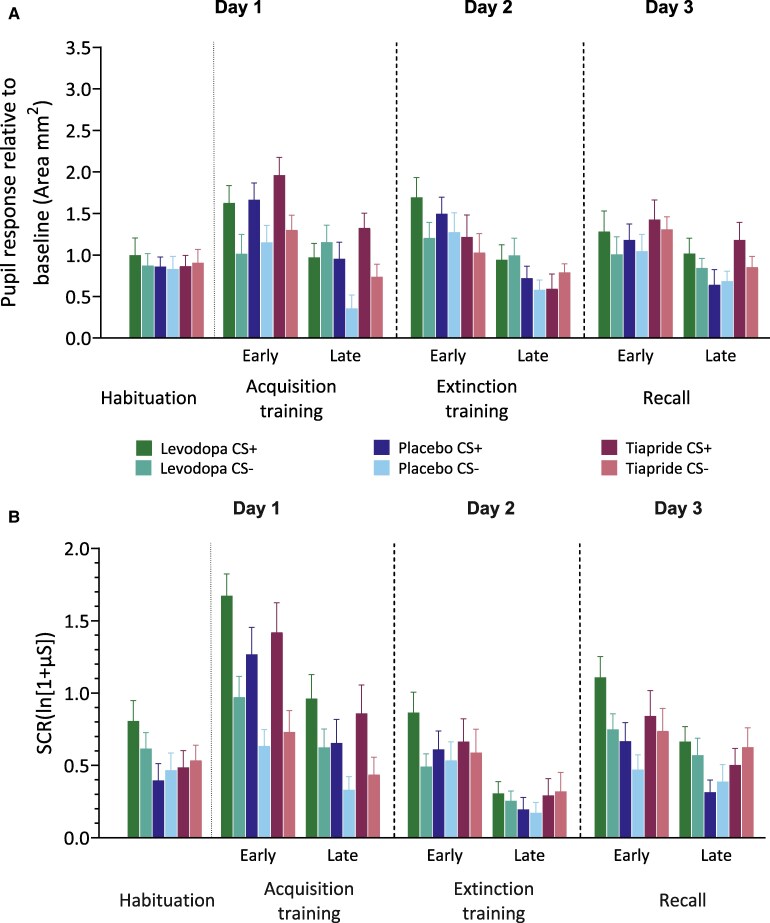
**Pupil and SCRs across phases in levodopa, tiapride and placebo groups.** (**A**) Pupil response relative to baseline for habituation and early and late blocks of acquisition and extinction training, as well as the recall phase in the groups receiving levodopa (*n* = 24), placebo (*n* = 24) or tiapride (*n* = 22). Bars represent the group means, and error bars indicate the standard error of the mean (SEM). During fear acquisition, the levodopa group showed significantly higher pupil responses to the late CS− compared with the placebo group (*P* = 0.010). No other significant drug–placebo differences were observed for pupil responses. (**B**) SCRs during habituation and early and late blocks of acquisition and extinction training, as well as the recall phase in the groups receiving levodopa (*n* = 24), placebo (*n* = 25) or tiapride (*n* = 22). Bars represent group means, error bars indicate SEM. During the habituation phase, SCR amplitude to CS+ was significantly higher in the levodopa group compared with placebo group (*P* = 0.012), and during recall, overall SCR amplitude was also significantly higher in the levodopa group compared with placebo group (*P* = 0.029). SCR and pupil data were analysed separately using non-parametric repeated-measures ATS. Each model included medication subgroup as a between-subjects factor and stimulus type (CS+ versus CS−) and block (early versus late phase) as within-subjects factors. Dunnett’s adjustment was applied when comparing treatment groups to placebo and Tukey’s correction was used for all other within-group comparisons. Only *P*-values from between-group comparisons (drug versus placebo) are reported in the figure legend.

During the habituation phase, the mean (differential) pupil size towards CS+ and CS− did not differ between groups (Fig. 2A). Non-parametric ATS revealed no significant main effects of Stimulus (*P* = 0.233), Group (*P* = 0.383) or Stimulus × Group (*P* = 0.613) interactions (Table S6).

#### Fear acquisition training

During fear acquisition training, levodopa, placebo and tiapride groups showed significantly higher pupil responses to CS+ compared with CS− and during the early compared with the late part of the phase (Fig. 2A). Non-parametric ATS revealed a significant main effect of Stimulus (*F*(1) = 37.40, *P*< 0.001) and Block (*F*(1) = 32.25, *P*< 0.001), but no Group difference (*P* = 0.296). Both Stimulus × Group (*F*(1.86) = 3.22, *P*< 0.044) and Stimulus × Group × Block (*F*(1.96) = 3.30, *P* < 0.038) interactions were significant, while other interactions (all *P* > 0.050) were not statistically significant (Table S6). Stimulus × Block × Group *post hoc* analysis demonstrated significantly higher pupil responses during early CS + compared with late CS + (*P* < 0.030), as well as in early CS+ compared with early CS− (*P* < 0.012) in the placebo group. The levodopa group exhibited significantly higher pupil responses following late CS− compared with the placebo group for late CS− (*P* = 0.010), with no difference observed for the CS+ stimuli. In comparison to the placebo group, no significant differences were observed for tiapride (all *P* > 0.530) regarding both CS+ and CS− pupil responses during early phase or late phase. Note, however, that for SCRs no significant group interaction effects were found in fear acquisition training.

#### Extinction training

During extinction training, levodopa, placebo and tiapride groups showed significantly higher pupil responses during the early compared with the late part of the phase and in particular towards the CS+ compared with the CS− (Fig. 2A). Non-parametric ATS revealed a significant main effect of Block (*F*(1) = 33.80, *P* < 0.001), but no Stimuli (*P* = 0.365) or Group difference (*P* = 0.060; Table S6). Stimulus × Block (*F*(1) = 5.50, *P* = 0.019) interaction demonstrated significantly higher pupil responses to CS+ in early compared with late blocks (all *P* < 0.001), other interactions (all *P* > 0.300) were not significant.

#### Recall

During recall, all three groups demonstrated significantly higher pupil size response during the early compared with the late part of the phase (Fig. 2A). There was no significant difference comparing groups or stimulus types. Non-parametric ATS revealed a main effect of early compared with late blocks (*F*(1) = 15.19, *P* < 0.001), while no main effects were observed for Stimulus type (*P* = 0.330) and Group (*P* = 0.130; Table S6). Additionally, none of the interactions were significant [Stimulus × Block (*P* = 0.950), Group × Stimulus (*P* = 0.879), Group × Block (*P* = 0.473) or Group × Stimulus × Block (*P* = 0.562)]. Likewise, reanalysis considering the first trial of recall only revealed no significant group differences (Fig. S2A; Table S7).

### Skin conductance responses (SCRs)

#### Habituation phase

During the habituation phase, the mean SCR amplitudes towards CS+ were higher in levodopa compared with placebo groups (Fig. 2B). Non-parametric ATS revealed no significant main effects of Stimulus (*P* = 0.490) and Group (*P* = 0.076), but a significant effect of Stimulus × Group (*P* = 0.002) interaction (Table S6). *Post hoc* analysis of Stimulus × Group demonstrated no significantly higher SCR amplitude for both CS+ and CS− between all three groups (all *P* > 0.088), except between levodopa CS+ and Placebo CS + (*P* = 0.012) of this phase.

#### Fear acquisition training

During fear acquisition training, levodopa, placebo and tiapride groups showed significantly higher mean SCR amplitudes to CS+ compared with CS− and during the early compared with the late part of the phase (Fig. 2B). Non-parametric ATS revealed a significant main effect of Stimulus (*F*(1) = 65.10, *P* < 0.001) and Block (*F*(1) = 59.45, *P* < 0.001), but no Group difference (*P* = 0.066). Stimulus × Block (*F*(1) = 6.17, *P* = 0.013) interaction was significant; other interactions (all *P* > 0.833) were not significant (Table S6). *Post hoc* analysis of Stimulus × Block demonstrated significantly higher CS+ compared with CS− SCR amplitude in both early and late (all *P* < 0.001) as well as higher CS+ and CS− SCR amplitude in early compared with late blocks (all *P* < 0.001).

#### Extinction training

During extinction training, levodopa, placebo and tiapride groups showed significantly higher mean SCR amplitudes towards the CS+ compared with the CS− and during the early compared with the late part of the phase (Fig. 2B). Non-parametric ATS revealed a significant main effect of Stimulus (*F*(1) = 11.08, *P* < 0.001) and Block (*F*(1) = 65.60, *P* < 0.001), but no Group difference (*P* = 0.158). Stimulus × Block (*F*(1) = 4.75, *P* = 0.029) interaction showed significant differences; other interactions (all *P* > 0.553) were not significant (Table S6). Post-hoc analysis of Stimulus × Block demonstrated significantly higher CS + compared with CS− SCR amplitude in early block only (*P* < 0.001; late block *P* = 0.711) as well as higher CS+ and CS− SCR amplitude in early compared with late blocks (all *P* < 0.001).

#### Recall

During recall, all three groups demonstrated significantly higher SCR amplitude during the early compared with the late part of the phase. The levodopa group demonstrated significantly higher SCR compared with the placebo group, but no significant difference comparing stimulus types (CS+ or CS; Fig. 2B). Non-parametric ATS revealed a significant main effect of Block (*F*(1) = 34.23, *P* < 0.001) and Group (*F*(1.99) = 3.00, *P* < 0.050), but no Stimulus difference (*P* = 0.121). The Stimulus × Block (*F*(1) = 7.6, *P* = 0.006) interaction was significant; all other interactions (all *P* > 0.180) were not significant (Table S6). Post-hoc analysis of Stimulus × Block interactions demonstrated significantly higher CS+ compared with CS− SCR amplitude in early block only (*P* = 0.016; late block *P* = 0.994) as well as higher CS+ and CS− SCR amplitude in early compared with late blocks (all *P* < 0.008). Furthermore, overall, SCR amplitude was significantly higher during the task for the levodopa group compared with the placebo group (*P* = 0.029), but not for the tiapride group compared with the placebo (*P* = 0.275) or the levodopa group (*P* = 0.581). Reanalysis considering the first trial of recall only revealed no significant group differences (Fig. S2B; Table S7).

## Results Group B (bromocriptine, placebo and haloperidol)

### Questionnaires

Across all Group B participants, CS+ was rated significantly higher in unpleasantness (valence), arousal and fear compared with CS− following fear acquisition training. Although this difference remained statistically significant during extinction and recall, CS+ ratings decreased over time, reflecting successful extinction learning (Fig. S3; Table S8; Table S9). Participants estimated a high likelihood of US occurrence following CS+ (96.40 ± 12.80%), while the probability was minimal for CS− (3.87 ± 15.24%; Table S5*)*. Valence, fear, arousal and US expectancy ratings are detailed in the supplement, along with information on CS–US contingency awareness and US unpleasantness (Fig. S3; Table S5; Table S8; Table S9).

### Pupillometry

#### Habituation phase

**Figure 3:**
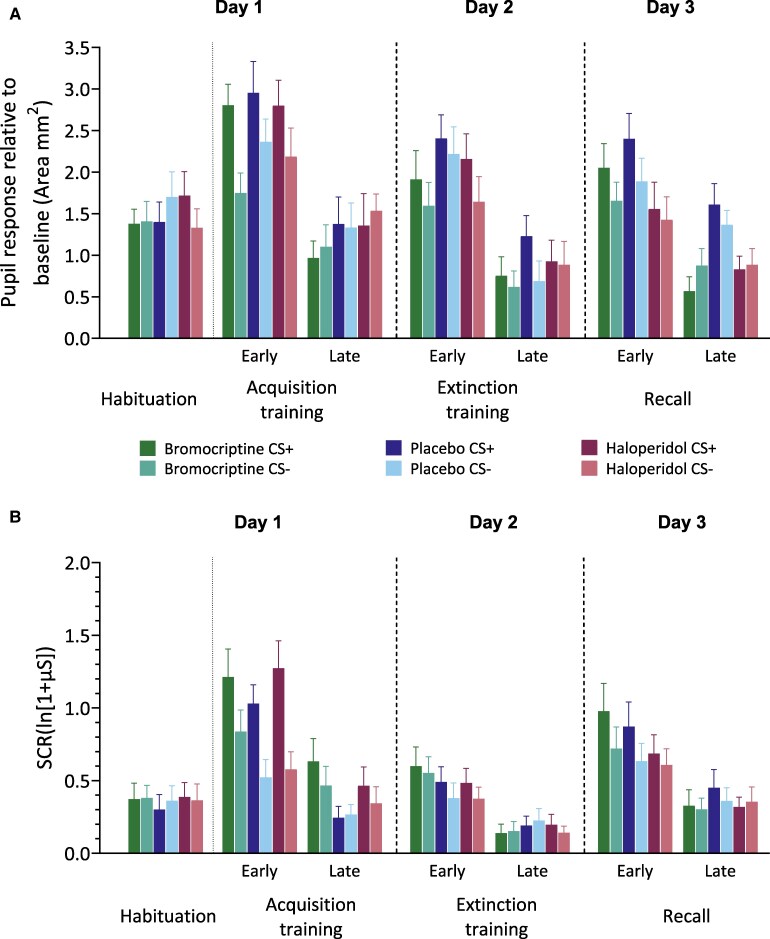
**Pupil and SCRs across phases in bromocriptine, haloperidol and placebo groups.** (**A**) Pupil responses relative to baseline for habituation and early and late blocks of fear acquisition and extinction training, as well as the recall phase in the groups receiving bromocriptine (*n* = 24), placebo (*n* = 25) or haloperidol (*n* = 25). Bars represent the group means, and error bars indicate the SEM. During recall, pupil responses were significantly lower in the bromocriptine group compared with placebo (*P* = 0.009), and in the haloperidol group compared with placebo group (*P* = 0.014) during the late block. No other significant between-group differences in pupil size were found. (**B**) SCRs during habituation and early and late blocks of fear acquisition and extinction training, as well as the recall phase in the groups receiving bromocriptine (*n* = 25), placebo (*n* = 25) or haloperidol (*n* = 24). Bars represent group means, error bars indicate SEM. No significant between-group differences in SCR amplitude were observed during any phase. SCR and pupil data were analysed separately using non-parametric repeated-measures ATS. Each model included medication subgroup as a between-subjects factor, and stimulus type (CS+ versus CS−) and block (early versus late phase) as within-subjects factors. Dunnett’s adjustment was applied when comparing treatment groups to placebo and Tukey’s correction was used for all other within-group comparisons. Only *P*-values from between-group comparisons (drug versus placebo) are reported in the figure legend.

During the habituation phase, the mean (differential) pupil size towards CS+ and CS− did not differ between groups. Non-parametric ATS revealed no significant main effects of Stimulus (*P* = 0.377), Group (*P* = 0.643) or Stimulus × Group (*P* = 0.626) interactions (Fig. 3A;  Table S10).

#### Fear acquisition training

During fear acquisition training, bromocriptine, placebo and haloperidol groups showed significantly higher pupil responses to CS+ compared with CS− and during the early compared with the late part of the phase (Fig. 3A). Non-parametric ATS revealed a significant main effect of Stimulus (*F*(1) = 6.84, *P* < 0.009) and Block (*F*(1) = 62.51, *P* < 0.001), but no Group difference (*P* = 0.602). Stimulus × Block (*F*(1) = 15.36, *P* < 0.001) interaction showed significant differences; other interactions (all *P* > 0.105) were not significant (Table S10). Post-hoc analysis of Stimulus × Block demonstrated significantly higher CS + and CS− pupil responses in early compared with late blocks (all *P* < 0.001) and higher pupil responses for CS + than for CS− trials in early (*P* < 0.001) but not late blocks (*P* = 0.978).

#### Extinction training

During extinction training, bromocriptine, placebo and haloperidol groups showed significantly higher pupil responses to CS+ compared with CS− and during the early compared with the late part of the phase (Fig. 3A). Non-parametric ATS revealed a significant main effect of Stimulus (*F*(1) = 6.10, *P* < 0.014) and Block (*F*(1) = 69.26, *P* < 0.001), but no Group difference (*P* = 0.248). Additionally, none of the interactions were significant [Stimulus × Block (*P* = 0.487), Group × Stimulus (*P* = 0.560), Group × Block (*P* = 0.377) or Group × Stimulus × Block (*P* = 0.278); Table S10].

#### Recall

During recall, bromocriptine, placebo and haloperidol groups demonstrated significantly higher pupil size response during the early compared with the late part of the phase and significant differences between both groups and the placebo group, but no significant difference between stimulus types (CS+ or CS−; Fig. 3A). Non-parametric ATS revealed a main effect of group (*F*(1.98) = 4.98, *P* = 0.007) and early compared with late blocks (*F*(1) = 48.97, *P* < 0.001), but no main effects for stimulus (*P* = 0.716, Table S10). The Block × Group (*F*(1.9) = 3.04, *P* = 0.050) interaction was significant; all other interactions (all *P* > 0.070) were not significant (Table S10). *Post* hoc analysis of the Block × Group interaction revealed significantly higher pupil responses in the early phase compared with the late phase for bromocriptine (*P* < 0.001), placebo (*P* < 0.009) and haloperidol groups (*P* < 0.014). Pupil responses during the early block did not differ significantly between groups (all *P* > 0.08). However, during the late block, the pupil responses were lower in the bromocriptine compared with the placebo group (*P* = 0.009), and in the haloperidol compared with the placebo group (*P* = 0.014). Reanalysis considering the first trial of recall only revealed no significant group differences (Fig. S4A; Table S11).

### Skin conductance responses (SCRs)

#### Habituation phase

During the habituation phase, the mean SCR amplitudes towards CS+ and CS− did not differ between groups (Fig. 3B). Non-parametric ATS revealed no significant main effects of Stimulus (*P* = 0.387), Group (P = 0.876) or Stimulus × Group (*P* = 0.349) interactions (Table S10).

#### Fear acquisition training

During fear acquisition training, bromocriptine, placebo and haloperidol groups showed significantly higher mean SCR amplitudes to CS+ compared with CS− and during the early compared with the late part of the phase (Fig. 3B). Non-parametric ATS revealed a significant main effect of Stimulus (*F*(1) = 31.17, *P* < 0.001) and Block (*F*(1) = 70.96, *P* < 0.001), but no Group effect (*P* = 0.741). Both Stimulus × Block (*F*(1) = 17.95, *P* < 0.001) and Stimulus × Group × Block (*F*(1.87) = 5.00, *P* < 0.008) interactions showed significant differences; all other interactions (all *P* > 0.410) were not significant (Table S10). Stimulus × Block × Group *post hoc* analysis revealed significantly higher SCR amplitudes during early CS+ compared with late CS+ for all three groups (all *P* < 0.006), as well as in the early CS+ compared with early CS− (all *P* < 0.016), but not during late part of the phase (all *P* > 0.26). In both the bromocriptine and placebo groups, early CS− exhibited significantly higher SCR amplitudes than late CS− (all *P* < 0.007). The haloperidol group did not show a significant decrease in SCR amplitude levels between early and late CS− (*P* = 0.503). Compared with the placebo group, no significant differences were observed for haloperidol (all *P* > 0.670) and bromocriptine (all *P* > 0.867) regarding both CS+ and CS− SCR amplitudes during either early or late parts of the phase.

#### Extinction training

During extinction training, all three groups demonstrated significantly higher SCR amplitude during the early compared with the late part of the phase with no significant differences between groups and group stimulus types (CS+ versus CS−) (Fig. 3B). Non-parametric ATS revealed a significant main effect of block (*F*(1) = 32.73, *P* < 0.001), while no main effects were observed for Stimulus (*P* = 0.280) and Group (*P* = 0.916). Additionally, none of the interactions [Stimulus × Block (*P* = 0.302), Group × Stimulus (*P* = 0.860), Group × Block (*P* = 0.218) and Group × Stimulus × Block (*P* = 0.699)] were significant (Table S10).

#### Recall

During recall, all three groups demonstrated significantly higher SCR amplitude during the early compared with the late part of the phase. There was no significant difference comparing groups or stimulus types (CS+ or CS−; Fig. 3B). Non-parametric ATS revealed a significant main effect of block (*F*(1) = 57.08, *P* < 0.001), while no main effects were observed for Stimulus (*P* = 0.100) and Group (*P* = 0.960). Additionally, none of the interactions were significant [Stimulus × Block (*P* = 0.114), Group × Stimulus (*P* = 0.682), Group × Block (*P* = 0.291) or Group × Stimulus × Block (*P* = 0.594); Table S10]. Reanalysis considering the first trial of recall only revealed no significant group differences (Fig. S4A; Table S11).

### Baseline

**Figure 4:**
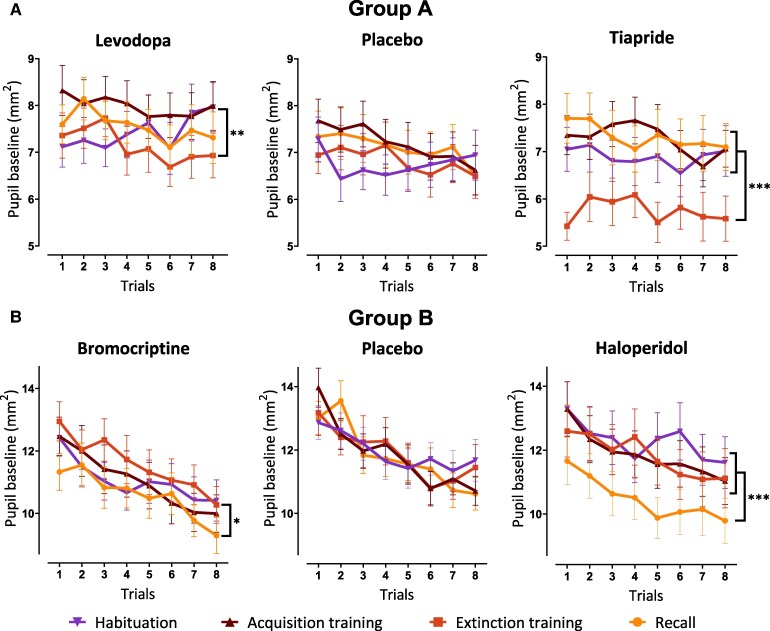
**Baseline pupil size across phases in all drug and placebo groups.** Pupil size variation (in mm^2^) 2 s prior to CS onset during the first 8 trials in both (**A**) Group A receiving levodopa (*n* = 24), placebo (*n* = 24) or tiapride (*n* = 22) and (**B**) Group B receiving bromocriptine (*n* = 24), placebo (*n* = 25) or haloperidol (*n* = 25). All data are presented as means, and error bars indicate the SEM. Non-parametric ATS were conducted separately within each drug group to assess baseline pupil size differences across phases. All *P*-values are adjusted using the Tukey–Kramer method for multiple comparisons (* *P* < 0.05; ** *P* < 0.01; *** *P* < 0.001).

In the baseline pupil size analysis (prestimulus, that is, 2 s prior to CS onset; Fig. 4), the placebo groups (A and B) showed no significant differences between phases (*P* = 0.357 for Group A, *P* = 0.571 for Group B). DA agonists, levodopa and bromocriptine, showed significant differences between only two phases: levodopa had a higher baseline during fear acquisition compared with extinction (*F*(2.99) = 4.38, *P* = 0.004), and bromocriptine had a higher baseline during extinction compared with recall (*F*(3) = 3.03, *P* = 0.028; Table S12). These limited differences suggest that the effects in the DA agonist groups may not be directly attributed to the drug. In contrast, DA antagonists tiapride and haloperidol showed significant decreases in baseline pupil size during one phase compared with all others: tiapride during extinction (*F*(3) = 22.94, *P* < 0.001) and haloperidol during recall (*F*(3) = 5.64, *P* < 0.001). These reductions in pupil size corresponded with the presence of the drugs in the blood, indicating intrinsic effects of the DA antagonists. For tiapride, which has a short half-life, the reduction in pupil size is observed during extinction training (Table S2). For haloperidol, the longer half-life of haloperidol, evidenced by the higher drug concentrations in the blood during recall compared with extinction, likely explains this observation (Table S2). Note that no bromocriptine level was measured on Day 2, likely due to the sensitivity limitations of the available test (detection limit: 0.1 ng/ml).

### Exploratory analyses

Finally, we conducted three exploratory analyses. Firstly, we tested whether serum drug concentrations correlated with differential CRs but found no significant associations after correcting for multiple comparisons (Table S13). Secondly, we assessed whether baseline depression, anxiety or stress scores (DASS-21-G) predicted fear learning outcomes. Correlations were low and non-significant, likely due to the study inclusion criteria (Table S14). Third, we investigated whether drug effects during recall differed in participants with successful extinction (CS+ ≤ CS− during late extinction) but found no significant drug × extinction success interactions. All are reported in the Supplement Exploratory section.

## Discussion

The main aim of the study was to explore the effect of dopaminergic agonists and antagonists on extinction learning and subsequent extinction and recall in humans, as measured by changes in autonomic CRs (SCR and pupil size) and self-reported questionnaire outcomes. Findings provide some additional support that the DA system is involved in fear extinction learning. Drug effects, however, were small and unspecific drug effects were also observed.

### Dopaminergic effects on fear extinction learning

We tested the hypothesis that pharmacological intake of different dopaminergic drugs (levodopa and bromocriptine) prior to extinction would enhance fear extinction learning, leading to a reduction in fear recall the following day. We were thus expecting to observe a diminution of SCR and pupil size CRs during both extinction training and the recall phase for both levodopa and bromocriptine groups compared with their respective placebo. However, although we found effects of dopaminergic drugs, findings were present during recall only, and effects following bromocriptine and levodopa intake were opposing.

In line with our hypothesis, we measured reduced pupil dilation during late recall in the bromocriptine group compared with the placebo group, indicating faster extinction of spontaneously recovered fear reactions on the third day. This could suggest that bromocriptine, as a dopaminergic agonist acting on postsynaptic DA D2 receptors, may facilitate fear extinction consolidation, thereby allowing for faster re-extinction on the following day. This aligns with previous findings where single doses of bromocriptine (1.25 or 2.5 mg) have demonstrated improvement in learning and memory formation in reversal learning, working memory and cognitive flexibility.^24,36,49,50^

On the other hand, contrary to our expectations, the levodopa group showed significantly higher SCR amplitudes during spontaneous recovery compared with the placebo group. This unexpected result may be explained by the intrinsic individual differences in SCR arousal levels,^45^ as supported by the fact that during the habituation phase, before any drug intake, participants in levodopa group already demonstrated higher overall SCR amplitudes. Furthermore, findings from Andres *et al*.^51^ suggest that the effect of levodopa in enhancing extinction memory consolidation may be favoured by environmental stress/arousal induced by an MRI. Indeed, they reported pronounced levodopa effects during experiments conducted within MRI environments.^22,25^ However, when experiments were behavioural only, conditional effects of levodopa were observed on SCR measures only in selected participants who had demonstrated successful extinction learning.^26^ Beyond these behavioural measures, Sartori *et al*.’s (2024)^52^ study suggests that levodopa effects on extinction are short unless DA reaches a sufficient level in the infralimbic cortex (IL). Using a mouse model with an impaired fear extinction mutation, they found that direct IL DA infusion is more effective than systemic administration, implying that levodopa’s overall interaction across multiple regions of the brain might contradict its impact on extinction.

### Anti-dopaminergic effects on fear extinction learning

We also investigated whether administering distinct anti-dopaminergic drugs (tiapride and haloperidol) prior to extinction would hinder fear response modulation during both fear extinction training and subsequent recall. Following the same logic, we anticipated less reduction of SCR and pupil size CRs during both extinction training and recall for both tiapride and haloperidol groups. However, unexpectedly, tiapride intake did not seem to affect fear extinction, and haloperidol demonstrated a decrease in pupil CR in late recall.

Additionally, contrary to our expectations, the group that took haloperidol showed no difference in SCRs and a diminished pupillary reaction during the late recall phase compared with its placebo group, suggesting a potential reduction in CRs. This is surprising given previous findings by Holtzman-Assif *et al*.^18^ showing that haloperidol-injected rats displayed increasing freezing behaviour compared with a control group during extinction sessions and on a drug-free recall test, indicating an impairment in fear response inhibition. Moreover, this effect was particularly pronounced when haloperidol was infused in the NAc, underscoring its critical role in fear extinction learning.

Interestingly, both DA antagonists appeared to have an intrinsic effect on the baseline tonic pupil size measure prior to each stimulus. We found that tiapride intake resulted in a reduction of baseline pupil size levels during extinction training compared to habituation, fear acquisition training and recall. Similarly, haloperidol intake led to a reduction of baseline levels during the recall phase compared to habituation, fear acquisition training and extinction training. Given that the drug is administered before extinction training, the reduction in baseline observed during recall but not extinction training following haloperidol intake may initially seem counterintuitive. However, this could be attributed to the prolonged half-life of haloperidol. The analysis of blood concentrations confirmed a higher presence of haloperidol in the blood on Day 3 compared with Day 2, all the other drugs were only detected on Day 2. According to the literature, the tonic pupil size impacts the magnitude of the following pupil responses.^29^ The effect of tiapride and haloperidol intake on tonic pupil size might explain the unexpected decrease in pupillary CRs, contrary to the anticipated increase.

### Limitations

The present study shows that assessing the dopaminergic system in healthy human participants by administration of oral dopaminergic and anti-dopaminergic drugs has limitations. One of the challenges in understanding the involvement of DA in fear extinction learning in humans is based on the widespread distribution of DA receptors across multiple areas of the brain. Each region, from the VTA to the NAc, including amygdala, hippocampus and prefrontal cortex, is thought to play a part in fear extinction, with DA likely exerting differential effects depending on the receptor subtype, dose and specific neural circuit involved. By orally administering drugs that modulate the overall DA levels, contradictory effects may be observed in brain regions containing DA receptors,^53^ leading to a result that is not clearly enhancing or impairing fear extinction learning.

Although the chosen timing and dosage of the respective drugs were based on existing literature, it did not consider individual variability. While it may be difficult and costly to try to evaluate individual's pharmacokinetics prior to the experiment, one could consider personalized dosing based on sex, age and weight. Furthermore, drug dosages may need to be higher to achieve significant effects. For example, based on the analytical technique used, we did not observe bromocriptine in blood samples taken after the extinction phase on Day 2.

Consistent with the existing literature, our study observed clear differential CRs for SCRs^29,31,32^ and pupil size.^29,31^ However, while SCRs, pupil responses and self-reported fear questionnaires can provide an overall assessment of fear acquisition and fear extinction success, it may not have been sufficient to measure the subtle change in CRs attributable to the effects of dopaminergic modulation. Future studies could benefit from brain imaging studies, in particular, combining PET/MRI^54^ to monitor DA release during extinction training and evaluate the changes due to systematic DA agonist or antagonist drug intake.

Finally, group sizes may have been too small. We observed differences between the groups already during habituation and fear acquisition training, that is, prior to drug intake, which is likely explained by individual variability in physiological measures.

### Summary and conclusions

In sum, bromocriptine intake resulted in faster re-extinction during recall, suggesting more robust consolidation of fear extinction. Contrary to our expectations, levodopa resulted in increased spontaneous recovery during early recall, possibly due to individual differences in SCR arousal levels. Regarding DA antagonists, tiapride showed no significant effects on fear extinction learning, while haloperidol unexpectedly led to faster re-extinction during late recall, potentially due to its prolonged half-life and intrinsic effects on tonic pupil size. Overall, while dopaminergic drugs show potential in modulating fear extinction and recall, providing support to the fact that the dopaminergic system contributes to extinction learning in humans, their effects are complex to interpret and seem to be influenced by multiple factors, including individual variability, drug receptor and environmental context.

## Supplementary Material

fcaf333_Supplementary_Data

## Data Availability

The data that support the findings of this study are available on request from the corresponding author. The code used for data processing and analysis is publicly available at: https://github.com/Spaldoub/code-doubliez-et-al-BrainComm-2025.
