# Supplementary material for: Dopaminergic drugs modulate fear extinction-related processes in humans, but effects are mild
Source: Brain Commun. 2025 Sep 8;7(5):fcaf333. doi: 10.1093/braincomms/fcaf333 (PMC12449160; doi:10.1093/braincomms/fcaf333)
Supplement: fcaf333_Supplementary_Data [file fcaf333_supplementary_data.pdf]

Supplementary: Dopaminergic drugs modulate fear extinction related processes in humans, but effects are mild

Short title: Dopamine drugs and fear extinction

Alice Doubliez[1], Kristina Köster*[1], Lara Müntefering*[1], Enzo Nio[1], Nicolas Diekmann[3], Andreas Thieme[1], Bilge Albayrak[2], Seyed Ali Nicksirat[1], Friedrich Erdlenbruch [1], Giorgi Batsikadze[1], Thomas Michael Ernst[1], Sen Cheng[3], Christian Josef Merz [4], Dagmar Timmann[1]

[1] Department of Neurology and Center for Translational Neuro- and Behavioral Sciences (C-TNBS), Essen University Hospital, University of Duisburg-Essen, Essen, Germany;

[2] Department of Pediatrics I and C-TNBS, Essen University Hospital, University of Duisburg-Essen, Essen, Germany;

[3] Institute for Neural Computation, Faculty of Computer Science, Ruhr University Bochum, Bochum, Germany;

[4]Department of Cognitive Psychology, Institute of Cognitive Neuroscience, Ruhr University Bochum, Bochum, Germany;

* Kristina Köster and Lara Müntefering contributed equally to this work.

Corresponding author

Alice Doubliez, MSc
Department of Neurology and Center for Translational Neuro- and Behavioral Sciences (C-TNBS)
Essen University Hospital, University of Duisburg-Essen
Hufelandstraße 55, Essen 45147, Germany
email: alice.doubliez@uk-essen.de
fax: +49 201 723 5534

Supplementary materials

Medication protocol and concentration 3
Supplementary materials – Group A 4
Questionnaires 4
CS valence, arousal, and fear ratings 5
US unpleasantness, CS/US contingency and US expectancy 5
Statistics for pupillometry and skin conductance responses 8
First recall trials pupillometry and skin conductance responses 9
Supplementary materials – Group B 10
Questionnaires 10
CS valence, arousal, and fear ratings 11
US unpleasantness, CS/US contingency and US expectancy 11
Statistics for pupillometry and skin conductance responses 13
First recall trials pupillometry and skin conductance responses 14
Exploratory analyses: Methods 15
Exploratory analyses: Results 16
Pupil size variation at baseline 16
Drug concentration and conditioned response correlations 16
DASS-21-G scores and conditioned response correlation 17
Fear recall in good and poor extinguishers across drug conditions 18

Medication protocol and concentration

Table S1 - Medication protocols of the different dopaminergic and anti-dopaminergic drugs day 2

GROUP A				GROUP B			
**Intake time**	**Levodopa**	**Placebo A**	**Tiapride**	**Intake time**	**Bromocriptine**	**Placebo B**	**Haloperidol**
0 min	Domperidone	Placebo	Tiapride	0 min	Domperidone	Placebo	Haloperidol
**60 min**	Levodopa	Placebo	Placebo	**30 min**	Bromocriptine	Placebo	Placebo
130 min	Beginning of the experiment			130 min	Beginning of the experiment		

Table S2 - Drug concentration measured each day in the participants blood after the experiment. * below detection limit 0.1 ng/mL. Measurements conducted by **MVZ Medizinisches Labor Bremen GmbH and by ***MVZ Dr. Eberhard & Partner Dortmund.

	Blood level		
Drugs	Day 1	Day 2	Day 3
Placebo A (n=25)	-	-	-
Levodopa (100 mg) (n=24) ***	-	0.66 ± 0.34 (µg/mL)	-
Tiapride (100 mg) (n=22) ***	-	0.54 ± 0.11 (µg/mL)	-
Placebo B (n=25)	-	-	-
Bromocriptine (1.25 mg) (n=25) **	-	*	-
Haloperidol (3 mg) (n=22) ***	-	0.24 ± 0.43 (ng/mL)	0.35 ± 0.22 (ng/mL)

Supplementary materials – Group A

Questionnaires

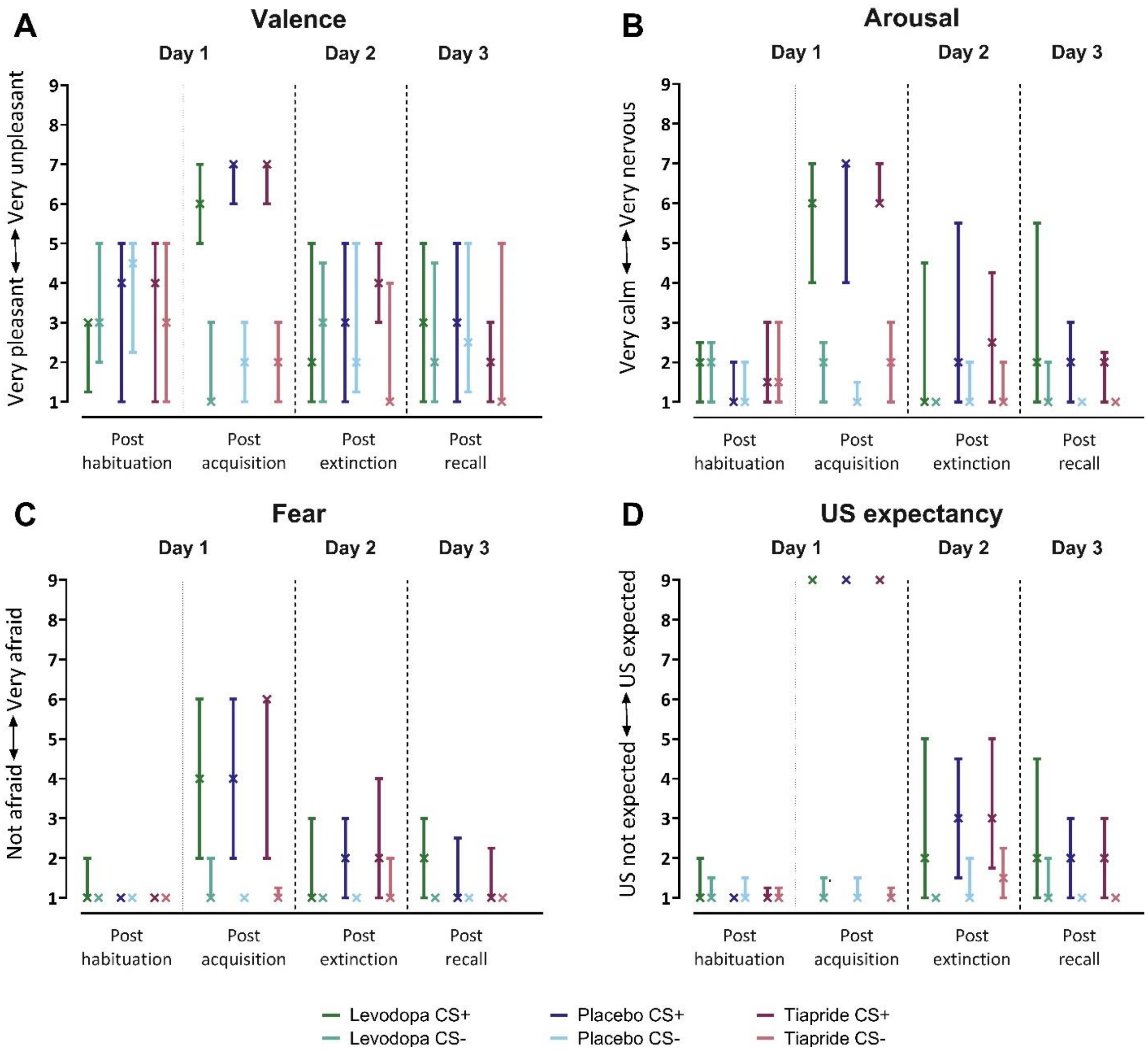

Figure S1 - **Self-reported fear evaluation questionnaire ratings in the groups receiving levodopa (n=24), placebo (n=25) or tiapride (n=22) post habituation, fear acquisition training, extinction training, and recall.** The median is marked by a cross (whiskers extend to the 1st and 3rd quartiles) for ratings of valence (A), arousal (B), fear (C), and US expectancy (D) based on a 1-9 Likert scale. Self-report data were analyzed using non-parametric ANOVA-type statistics for repeated measures. Rating type (valence, arousal, fear, US expectancy) served as the dependent variable. Stimulus type (CS+ vs. CS–) and phase (post-habituation, post-acquisition, post-extinction, and post-recall) were within-subject factors, and medication group was included as a between-subjects factor. After fear acquisition, CS+ was consistently rated as more unpleasant, arousing, fear-inducing, and predictive of the US than CS- (all $p<0.001$). These differences persisted through extinction and recall. No significant differences were observed during habituation (all $p>0.80$ for valence, arousal, fear; $p>0.96$ for US expectancy) or between drug groups (all $p>0.10$).

CS valence, arousal, and fear ratings

Following the habituation phase no significant differences in valence, arousal, and fear ratings between CS+ and CS- were present, and this was consistent across levodopa, placebo and tiapride groups. After fear acquisition training, CS+ received significantly higher unpleasantness ratings, higher arousal ratings, and was perceived as inducing more fear than CS- in all three groups (*Figure S1*). These distinctions between CSs persisted in every group during extinction and recall phases. Post acquisition training ratings consistently highlighted significant differences between CS+ and CS- in valence, arousal and fear ratings in all groups (*Table S3)*. Non-parametric ANOVA-type statistic revealed a significant main effect of Stimulus and Phase (main effects: all p<0.001), and a Stimulus × Phase interaction for arousal ($F_{(2.71)=}$40.30, p<0.001), valence ($F_{(2.51)=}$37.91, p<0.001) and fear ($F_{(2.69)=}$34.91, p<0.001) ratings independently of the drug group (*Table S4*). Post-hoc tests showed significant differences between stimuli post fear acquisition training, extinction training and recall phases (least square means tests, Valence: all p<0.001; Arousal: all p<0.001; Fear: all p<0.001), but not following the habituation phase (least square means test, all p>0.80). No significant differences in arousal, fear and valence ratings between groups were found.

US unpleasantness, CS/US contingency and US expectancy

Across all participants of the three groups, the likelihood that a US onset occurred after CS+ presentation was estimated to be 98.21 ± 11.40% (100% probability for 68 out of 71 participants), while after CS- presentation it was 1.54 ± 5.34% (0% probability for 69 out of 71 participants; *Table S5*). Overall median US unpleasantness was rated 7 (IQR 7-8) following acquisition, participants reported recognizing the association between CS+ and US after experiencing an average of 2.52 ± 1.63 electric shocks. No significant between groups for both US unpleasantness and CS/US contingency ratings have been found.

After habituation phase, reported US expectancy following CS+ and CS- were not significantly different from each other. Post fear acquisition training, participants reported a higher US expectancy after CS + compared to the CS- and this difference remained until the end of recall. Similarly to valence, arousal and fear ratings, post-acquisition training consistently showed significant differences between CS+ and CS- in US expectancy ratings in all groups (*Figure S1; Table S3*). Non-parametric ANOVA-type statistic revealed a significant main effect of Stimulus and Phase (main effects: all p<0.001), and a Stimulus × Phase interaction for US expectancy ($F_{(2.39)}$=136.24, p<0.001) ratings independently of the drug group (*Table S4*). Post-hoc tests showed significant differences between stimuli post fear acquisition training, extinction training and recall (least square means tests, US expectancy: all p<0.001), but not following habituation (least square means test, p>0.96).

Table S3 - **Self-reported fear evaluation questionnaire ratings in the groups receiving levodopa (Levo.), placebo (Plac.) or tiapride (Tiap.) post habituation, fear acquisition training, extinction training, and recall.** Median (interquartile range) ratings of valence (A), arousal (B), fear (C) and US expectancy (D) based on a 1-9 Likert scale. Statistically significant differences between CS+ and CS- are shown in bold (least square means tests, p<0.01). Note that no statistically significant differences were found between drug groups and placebo group.

Stimulus	Time of assessment											
	Post habituation			Post acquisition			Post extinction			Post recall		
	Levo.	Plac.	Tiap.	Levo.	Plac.	Tiap.	Levo.	Plac.	Tiap.	Levo.	Plac.	Tiap.
Valence ratings (1 – comfortable, 9 – uncomfortable)												
CS+	3 (1-3)	4 (1-5)	5 (1-5)	**6 (5-7)**	**7 (6-7)**	**7 (6-7)**	**2 (1-5)**	**3 (1-5)**	**4 (3-5)**	**3 (1-5)**	**3 (1-5)**	**2 (1-3)**
CS-	3 (2-5)	4 (2-5)	3 (1-5)	**1 (1-3)**	**2 (1-3)**	**2 (1-3)**	**3 (1-4)**	**2 (1-5)**	**1 (1-4)**	**2 (1-4)**	**3 (1-5)**	**1 (1-4)**
Arousal ratings (1 – very calm, 9 – very nervous)												
CS+	2 (1-2)	1 (1-2)	2 (1-3)	**6 (4-7)**	**7 (4-7)**	**6 (6-7)**	**1 (1-4)**	**2 (1-5)**	**3 (1-4)**	**2 (1-5)**	**2 (1-3)**	**2 (1-2)**
CS-	2 (1-2)	1 (1-2)	2 (1-3)	**2 (1-2)**	**1 (1-1)**	**2 (1-3)**	**1 (1-1)**	**1 (1-2)**	**1 (1-2)**	**1 (1-2)**	**1 (1-1)**	**1 (1-2)**
Fear ratings (1 – not afraid, 9 – very afraid)												
CS+	1 (1-2)	1 (1-1)	1 (1-1)	**4 (2-6)**	**4 (2-6)**	**6 (2-6)**	**1 (1-3)**	**2 (1-3)**	**2 (1-4)**	**2 (1-3)**	**1 (1-2)**	**1 (1-2)**
CS-	1 (1-1)	1 (1-1)	1 (1-1)	**1 (1-2)**	**1 (1-1)**	**1 (1-1)**	**1 (1-1)**	**1 (1-1)**	**1 (1-2)**	**1 (1-1)**	**1 (1-1)**	**1 (1-1)**
US expectancy ratings (1 – US not expected, 9 – US surely expected)												
CS+	1 (1-2)	1 (1-1)	1 (1-1)	**9 (9-9)**	**9 (9-9)**	**9 (9-9)**	**2 (1-5)**	**3 (2-4)**	**3 (2-5)**	**2 (1-4)**	**2 (1-3)**	**2 (1-3)**
CS-	1 (1-1)	1 (1-1)	1 (1-1)	**1 (1-1)**	**1 (1-1)**	**1 (1-1)**	**1 (1-1)**	**1 (1-2)**	**2 (1-2)**	**1 (1-2)**	**1 (1-1)**	**1 (1-1)**

Table S4 - **Self-reported fear evaluation questionnaire ratings in the groups receiving levodopa, placebo or tiapride.** Results of the non-parametric ANOVA-type statistics for repeated measures on all phases. (* p<0.05; ** p<0.01; *** p<0.001).

Factor	Num DF	Den DF	F	Pr>F(infty)
Valence				
Phase	2.49	72.2	16.38	**<.001 *****
Stimulus	1	57.3	29.59	**<.001 *****
Group	1.98	67.5	0.41	0.663
Stimulus * Phase	2.51	76.2	37.91	**<.001 *****
Group * Phase	4.6	72.2	0.78	0.554
Stimulus * Group	1.99	57.3	0.84	0.430
Stimulus * Group * Phase	4.66	76.2	0.37	0.856
Arousal				

Phase	2.5	154	26.80	<.001 ***
Stimulus	1	67.9	162.16	<.001 ***
Group	1.99	67	0.54	0.584
Stimulus * Phase	2.71	164	40.30	<.001 ***
Group * Phase	4.78	154	1.62	0.155
Stimulus * Group	2	67.9	1.15	0.318
Stimulus * Group * Phase	5.16	164	0.34	0.896
		Fear		
Phase	2.74	171	29.76	<.001 ***
Stimulus	1	67.4	146.39	<.001 ***
Group	2	67.9	0.13	0.881
Stimulus * Phase	2.69	153	34.91	<.001 ***
Group * Phase	5.25	171	1.50	0.183
Stimulus * Group	1.99	67.4	0.14	0.865
Stimulus * Group * Phase	4.97	153	0.50	0.773
		US Expectancy		
Phase	2.7	204	39.29	<.001 ***
Stimulus	1	64.9	374.69	<.001 ***
Group	1.91	101	0.30	0.727
Stimulus * Phase	2.39	155	136.24	<.001 ***
Group * Phase	5.15	204	1.10	0.356
Stimulus * Group	1.96	64.9	0.18	0.831
Stimulus * Group * Phase	4.64	155	0.64	0.660

Table S5 - **Fear conditioning questionnaires on US in both group A receiving levodopa, placebo or tiapride and group B receiving bromocriptine, placebo or haloperidol.** Median (interquartile range) of US expectancy ratings and mean percentage of US perception following each CS and number of US perceived before CS/US contingency recognition post fear acquisition. Note that no statistically significant differences were found between drug groups and placebo group.

	**Post acquisition**					
	Levodopa	Placebo	Tiapride	Bromocriptine	Placebo	Haloperidol
	US unpleasantness (1 – US not unpleasant, 9 – US highly unpleasant)					
US	7 (6-8)	7 (7-8)	8 (7-8)	8 (7-8)	7 (6-8)	8 (7-8)
	Estimated probability of CS followed by US (%)					
CS+	100.00<br>100 % for 24/24	96.80 ± 16.00<br>100 % for 24/25	97.73 ± 8.69<br>100 % for 20/22	97.20 ± 14.00<br>100 % for 24/25	94.80 ± 14.18<br>100 % for 21/25	97.20 ± 10.21<br>100 % for 22/25
CS-	0.00<br>0% for 24/24	0.40 ± 2.00<br>0% for 24/25	2.27 ± 8.69<br>0% for 20/22	1.20 ± 6.00<br>0% for 24/25	8.00 ± 22.55<br>0% for 22/25	2.40 ± 12.00<br>0% for 24/25
	Number of US perceived before CS/US contingency recognition					
CS/US	2.80 ± 1.38	2.2 ± 0.96	2.59 ± 1.84	2.13 ± 0.80	2.38 ± 1.53	2.30 ± 1.82

Statistics for pupillometry and skin conductance responses

These methods use ANOVA-type statistic with the denominator degrees of freedom set to infinity (Brunner et al., 2002[1]; Noguchi et al., 2012[2]) to enhance the reliability of the ANOVA-type statistic. Using finite denominator degrees of freedom can lead to increased type I errors (Bathke et al., 2009[3]).

Table S6 – **Pupil size and skin conductance responses statistics for the groups receiving levodopa, placebo or tiapride.** Results of the non -parametric ANOVA-type statistics for repeated measures on all phases. (* p<0.05; ** p<0.01; *** p<0.001)

Pupillometry

Factor	Num DF	Den DF	F	Pr>F(nifty)
Habituation				
Stimulus	1	60.4	1.42	0.233
Group	1.95	61.4	0.95	0.383
Stimulus * Group	1.84	60.4	0.46	0.613
Fear Acquisition training				
Block	1	66.7	32.25	<.001 ***
Stimulus	1	55.5	37.40	<.001 ***
Group	1.96	61.5	1.22	0.296
Stimulus * Block	1	65.1	1.48	0.224
Group * Block	1.95	66.7	2.96	0.053
Stimulus * Group	1.86	55.5	3.22	0.044 *
Stimulus*Group*Block	1.96	65.1	3.30	0.038 *
Extinction training				
Block	1	61.3	33.80	<.001 ***
Stimulus	1	62.2	0.82	0.365
Group	2	64.5	2.82	0.060
Stimulus * Block	1	63.1	5.50	0.019 *
Group * Block	1.96	61.3	0.95	0.384
Stimulus * Group	1.89	62.2	1.05	0.346
Stimulus*Group*Block	1.99	63.1	0.17	0.843
Recall				
Block	1	52.2	15.19	<.001 ***
Stimulus	1	62.1	0.95	0.330
Group	1.99	63.5	2.04	0.130
Stimulus * Block	1	55.6	0.00	0.950
Group * Block	1.84	52.2	0.73	0.473
Stimulus * Group	1.99	62.1	0.13	0.879
Stimulus*Group*Block	1.88	55.6	0.56	0.562

SCR

Factor	Num DF	Den DF	F	Pr>F(nifty)
Habituation				
Stimulus	1	59.8	0.48	0.490
Group	1.99	67.1	2.58	0.076
Stimulus * Group	1.91	59.8	6.46	0.002 **
Fear Acquisition training				
Block	1	62.1	59.45	<.001 ***
Stimulus	1	67	65.10	<.001 ***
Group	1.97	64.4	2.74	0.066
Stimulus * Block	1	65.1	6.17	0.013 *
Group * Block	1.93	62.1	0.14	0.859
Stimulus * Group	1.98	67	0.18	0.833
Stimulus*Group*Block	1.97	65.1	0.17	0.844
Extinction training				
Block	1	60	65.60	<.001 ***
Stimulus	1	58.6	11.08	<.001 ***
Group	1.94	63.2	1.85	0.158
Stimulus * Block	1	63.1	4.75	0.029 *
Group * Block	1.89	60	0.54	0.573
Stimulus * Group	1.86	58.6	0.28	0.741
Stimulus*Group*Block	1.9	63.1	0.57	0.554
Recall				
Block	1	65.5	34.23	<.001 ***
Stimulus	1	50.4	2.40	0.121
Group	1.99	67	3.00	0.050 *
Stimulus * Block	1	67.3	7.60	0.006 **
Group * Block	1.96	65.5	1.06	0.345
Stimulus * Group	1.75	50.4	1.74	0.180
Stimulus*Group*Block	1.99	67.3	0.63	0.529

First recall trials pupillometry and skin conductance responses

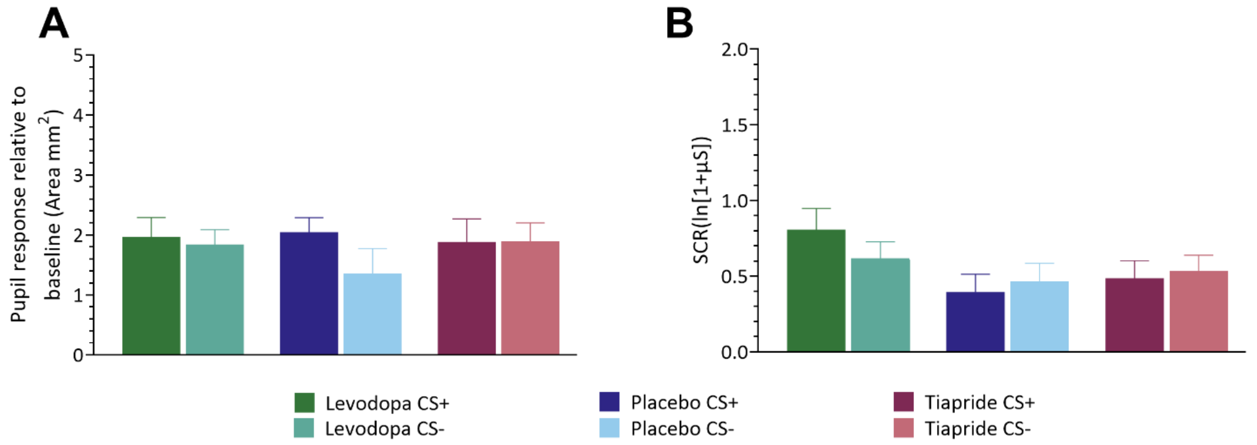

Figure S2 - (A) **Pupil responses relative to baseline for first recall trial in the groups receiving levodopa (n=21), placebo (n=23) or tiapride(n=22)**. (B) **Skin conductance responses (SCRs) for first recall trial in the groups receiving levodopa (n=24), placebo (n=25) or tiapride (n=22)**. Bars represent the group means, and error bars indicate the standard error of the mean (S.E.M). Separate non-parametric ANOVA-type statistics were conducted for pupil and SCR data, with medication subgroup as a between-subjects factor and stimulus type (CS+ vs. CS−) as a within-subjects factor. Post-hoc comparisons were performed using Dunnett's adjustment to compare each drug group to its respective placebo. A significant main effect of stimulus was observed for both pupil responses (p=0.041) and SCRs (p<0.005), indicating spontaneous recovery. No significant main effects of drug group were found (Pupil: p=0.451; SCR: p=0.127), and stimulus × group interactions were not significant (Pupil: p=0.230; SCR: p=0.081).

We assessed the first trial in recall across levodopa, placebo, and tiapride groups using both pupil dilation and SCR (*Figure S2 – A and B; Table S7*). Both pupil dilation and SCR showed a significant difference between CS+ and CS- trials (Pupil: $F_{(1)}$=4.19, p=0.045; SCR: p<0.006), indicating spontaneous recovery of the initial fear association. However, there were no significant differences between the drug groups for either measure (Pupil: $F_{(1.97)}$=0.79, p=0.451; SCR: $F_{(1.96)}$=2.07, p=0.127). For the interaction between CS type and drug group, neither measure showed a significant effect (Pupil: $F_{(1.95)}$=1.47, p=0.230; SCR: $F_{(1.91)}$=2.55, p=0.081; *Table S7*).

Table S7 - Pupillometry and SCR statistics in the groups receiving levodopa, placebo and tiapride. Results of the non-parametric ATS for repeated measures on the first trial of recall. (*p < 0.01; ** p < 0.05; *** p < 0.001). Denominator degrees of freedom were set to infinity

Factor	Num DF	Den DF	F	Pr>F(infty)
		Pupillometry		
Stimulus	1	55	4.19	0.041 *
Group	1.97	59.5	0.79	0.451
Stimulus * Group	1.95	55	1.47	0.230
		SCR		
Stimulus	1	60.1	8.07	0.005 **
Group	1.96	63.6	2.07	0.127
Stimulus * Group	1.91	60.1	2.55	0.081

Supplementary materials – Group B

Questionnaires

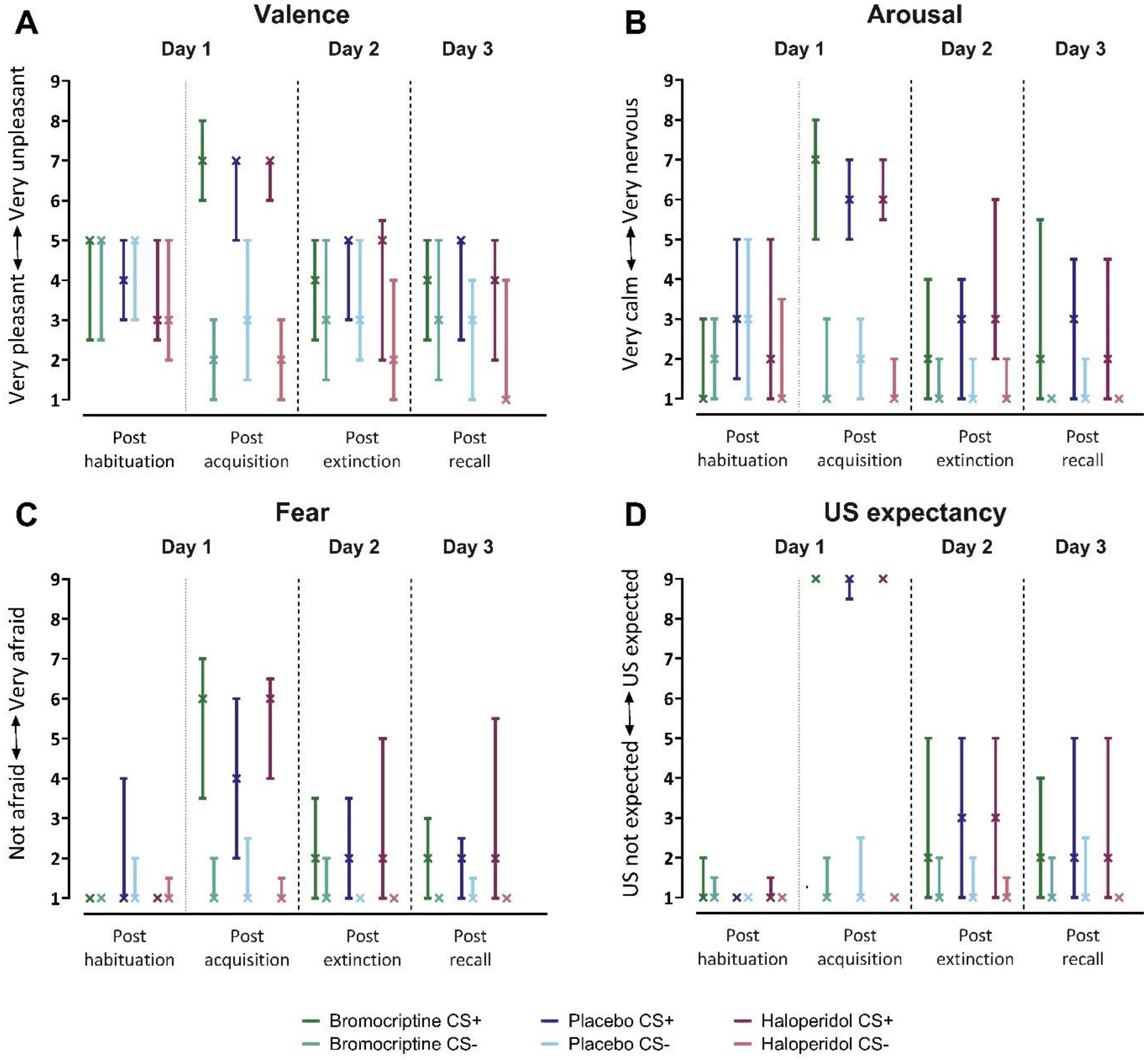

Figure S3 - **Self-reported fear evaluation questionnaire ratings in the groups receiving bromocriptine (n=25), placebo (n=25) or haloperidol (n=25) post habituation, fear acquisition training, extinction training, and recall.** The median is marked by a cross (whiskers correspond to the 1st and 3rd quartiles) for ratings of valence (A), arousal (B), fear (C), and US expectancy (D) based on a 1-9 Likert scale. Self-report data were analyzed using non-parametric ANOVA-type statistics for repeated measures. Rating type (valence, arousal, fear, US expectancy) served as the dependent variable. Stimulus type (CS+ vs. CS−) and phase (post-habituation, post-acquisition, post-extinction, and post-recall) were within-subject factors, and medication group was included as a between-subjects factor. After fear acquisition, CS+ was consistently rated as more unpleasant, arousing, fear-inducing, and predictive of the US than CS− (all $p < 0.001$). These differences persisted through extinction and recall. No significant CS+ vs. CS− differences were observed during habituation (valence, arousal, fear: all $p > 0.99$; US expectancy: $p > 0.98$), and no main effects

of drug group were found. A three-way interaction (Stimulus × Group × Phase, p = 0.021) was observed for fear ratings, but post-hoc tests did not reveal consistent or meaningful group-specific effects.

CS valence, arousal, and fear ratings

Post habituation phase, no significant differences in valence, arousal, and fear ratings between CS+ and CS- were present, and this was consistent across bromocriptine, placebo and haloperidol groups. Following fear acquisition training, CS+ received significantly higher unpleasantness ratings, higher arousal ratings, and was perceived as inducing more fear than CS- in all three groups (*Figure S3; Table S8*). These distinctions between CSs persisted in every group during extinction and recall phases. Post acquisition training ratings consistently highlighted significant differences between CS+ and CS- in valence, arousal and fear ratings in all groups. Non-parametric ANOVA-type statistic revealed a significant main effect of Stimulus and Phase (main effects: all p<0.001), and a Stimulus × Phase interaction for arousal ($F_{(2.66)=}$39.44, p<0.001), valence ($F_{(2.63)=}$80.49, p<0.001) and fear ($F_{(2.63)=}$41.18, p<0.001) ratings independently of the drug group, as well as a Stimulus x Group x Phase interaction in fear ratings ($F_{(4.92)}$=2.66, p=0.021; *Table S9*). Post-hoc tests showed significant differences between stimuli post fear acquisition training, extinction training and recall phases (least square means tests, Valence: all p<0.001; Arousal: all p<0.001; Fear: all p<0.001), but not following the habituation phase (least square means test, all p>0.99).

US unpleasantness, CS/US contingency and US expectancy

Across all participants of the three groups, the likelihood that a US occurred after CS+ presentation was estimated to be 96.40 ± 12.80% (100% probability for 67 out of 75 participants), while after CS- presentation it was 3.87 ± 15.24% (0% probability for 72 out of 75 participants; Figure S3; Table S5). Overall median US unpleasantness was rated 7 (IQR 7-8) following acquisition, participants reported recognizing the association between CS+ and US after experiencing an average of 2.27 ± 1.42 electric shocks. No significant differences between groups for both US unpleasantness and CS/US contingency ratings have been found. After the habituation phase, reported US expectancy following CS+ and CS- were not significantly different from each other. Post fear acquisition training, participants reported a higher US expectancy after CS + compared to the CS- and this difference remained until the end of recall (Figure S3; Table S8). Non-parametric ANOVA-type statistic revealed a significant main effect of Stimulus and Phase (main effects: all p< 0.001), and a Stimulus × Phase interaction for US expectancy ($F_{(2.74)}$=120.13, p<0.001) ratings independently of the drug group (Table S9). Post-hoc tests showed significant differences between stimuli post fear acquisition training, extinction training, and recall phase (least square means tests, US expectancy: all p<0.001), but not following habituation (least square means test, p>0.98).

Table S8 - Self-reported fear evaluation questionnaire ratings in the groups receiving bromocriptine (Brom.), placebo (Plac.) or haloperidol (Halo.) post habituation, fear acquisition training, extinction training, and recall. Median (interquartile range) ratings of valence (A), arousal (B), fear (C) and US expectancy (D) based on a 1-9 Likert scale. Statistically significant differences between CS+ and CS- are shown in bold (least square means tests, p<0.01). Note that no statistically significant differences were found between drug groups and placebo group.

Stimulus	Time of assessment											
	Post Habituation			Post acquisition			Post extinction			Post recall		
	Brom.	Plac.	Halo.	Brom.	Plac.	Halo.	Brom.	Plac.	Halo.	Brom.	Plac.	Halo.
Valence ratings (1 –comfortable, 9 – uncomfortable)												
CS+	5 (3-5)	4 (3-5)	3 (3-5)	**7 (6-8)**	**7 (5-7)**	**7 (6-7)**	4 (3-5)	**5 (3-5)**	**5 (2-5)**	**4 (3-5)**	**5 (2-5)**	**4 (2-5)**
CS-	5 (3-5)	5 (3-5)	3 (2-5)	**2 (1-3)**	**3 (2-5)**	**2 (1-3)**	3 (2-5)	**3 (2-5)**	**2 (1-4)**	**3 (2-5)**	**3 (1-4)**	**1 (1-3)**
Arousal ratings (1 – very calm, 9 – very nervous)												
CS+	1 (1-3)	3 (2-5)	2 (1-5)	**7 (5-8)**	**6 (5-7)**	**6 (6-7)**	**2 (1-4)**	**3 (1-4)**	**3 (2-6)**	**2 (1-5)**	**3 (1-4)**	**2 (1-4)**
CS-	2 (1-3)	3 (1-5)	1 (1-3)	**1 (1-3)**	**2 (1-3)**	**1 (1-2)**	**1 (1-2)**	**1 (1-2)**	**1 (1-2)**	**1 (1-1)**	**1 (1-2)**	**1 (1-1)**
Fear ratings (1 – not afraid, 9 – very afraid)												
CS+	1 (1-1)	1 (1-4)	1 (1-1)	**6 (4-7)**	**4 (2-6)**	**6 (4-6)**	**2 (1-3)**	**2 (1-3)**	**2 (1-5)**	**2 (1-3)**	**2 (1-2)**	**2 (1-5)**
CS-	1 (1-1)	1 (1-2)	1 (1-1)	**1 (1-2)**	**1 (1-2)**	**1 (1-1)**	**1 (1-2)**	**1 (1-1)**	**1 (1-1)**	**1 (1-1)**	**1 (1-1)**	**1 (1-1)**
US expectancy ratings (1 – US not expected, 9 – US surely expected)												
CS+	1 (1-2)	1 (1-1)	1 (1-1)	**9 (9-9)**	**9 (9-9)**	**9 (9-9)**	**2 (1-5)**	**3 (1-5)**	**3 (1-5)**	**2 (1-4)**	**2 (1-5)**	**2 (1-5)**
CS-	1 (1-1)	1 (1-1)	1 (1-1)	**1 (1-2)**	**1 (1-2)**	**1 (1-1)**	**1 (1-2)**	**1 (1-2)**	**1 (1-1)**	**1 (1-2)**	**1 (1-2)**	**1 (1-1)**

Table S9 - Self-reported fear evaluation questionnaire ratings in the groups receiving bromocriptine, placebo or haloperidol. Results of the non-parametric ATS for repeated measures on all phases. (* p<0.05; ** p<0.01; *** p<0.001)

Factor	Num DF	Den DF	F	Pr>F(infty)
Valence				
Phase	2.69	176	14.72	<.001 ***
Stimulus	1	67.2	126.10	<.001 ***
Group	1.96	69.1	0.93	0.392
Stimulus * Phase	2.63	168	80.49	<.001 ***
Group * Phase	5.12	176	0.76	0.583
Stimulus * Group	1.93	67.2	1.24	0.288
Stimulus * Group * Phase	4.94	168	1.67	0.140
Arousal				

Phase	2.72	179	28.06	<.001 ***
Stimulus	1	64.3	154.88	<.001 ***
Group	1.98	70.6	0.99	0.370
Stimulus * Phase	2.66	176	39.44	<.001 ***
Group * Phase	5.19	179	1.17	0.320
Stimulus * Group	1.89	64.3	1.12	0.325
Stimulus * Group * Phase	5.09	176	1.68	0.133
Fear				
Phase	2.49	155	32.53	<.001 ***
Stimulus	1	69.6	147.32	<.001 ***
Group	2	71.8	0.27	0.766
Stimulus * Phase	2.63	166	41.18	<.001 ***
Group * Phase	4.61	155	0.93	0.458
Stimulus * Group	1.97	69.6	0.44	0.644
Stimulus * Group * Phase	4.92	166	2.66	**0.021 ***
US Expectancy				
Phase	1.89	129	58.51	<.001 ***
Stimulus	1	70.4	299.07	<.001 ***
Group	1.98	70.7	0.28	0.755
Stimulus * Phase	2.74	195	120.13	<.001 ***
Group * Phase	3.68	129	1.27	0.280
Stimulus * Group	1.98	70.4	1.25	0.286
Stimulus * Group * Phase	5.45	195	0.59	0.721

Statistics for pupillometry and skin conductance responses

These methods use ANOVA-type statistic with the denominator degrees of freedom set to infinity (Brunner et al., 2002[1]; Noguchi et al., 2012[2]) to enhance the reliability of the ANOVA-type statistic. Using finite denominator degrees of freedom can lead to increased type I errors (Bathke et al., 2009[3]).

Table S10 - Pupil size and SCR statistics in the groups receiving bromocriptine, placebo or haloperidol. Results of the non-parametric ATS for repeated measures on all phases. (* p<0.05; ** p<0.01; *** p<0.001)

**Pupillometry**					**SCR**				
Factor	Num DF	Den DF	F	Pr>F(nifty)	Factor	Num DF	Den DF	F	Pr>F(nifty)
Habituation					Habituation				
Stimulus	1	63.1	0.78	0.377	Stimulus	1	70.4	0.75	0.387
Group	1.93	66.4	0.43	0.643	Group	1.99	69	0.13	0.876
Stimulus * Group	1.91	63.1	0.45	0.626	Stimulus * Group	1.98	70.4	1.05	0.349
Fear Acquisition training					Fear Acquisition training				
Block	1	63.4	62.51	<.001 ***	Block	1	70.1	70.96	<.001 ***
Stimulus	1	66.6	6.84	**0.009 ****	Stimulus	1	52.3	31.17	<.001 ***
Group	1.94	67.2	0.50	0.602	Group	1.87	62.9	0.28	0.741
Stimulus * Block	1	69.4	15.36	<.001 ***	Stimulus * Block	1	63	17.95	<.001 ***
Group * Block	1.89	63.4	0.76	0.460	Group * Block	1.97	70.1	0.13	0.873
Stimulus * Group	1.95	66.6	0.64	0.523	Stimulus * Group	1.68	52.3	0.84	0.415
Stimulus * Group * Block	1.98	69.4	2.26	0.105	Stimulus * Group * Block	1.87	63	5.00	**0.008 ****
Extinction training					Extinction training				

Block	1	68.5	69.26	<.001 ***
Stimulus	1	66.9	6.10	**0.014 ***
Group	1.96	66.6	1.39	0.248
Stimulus * Block	1	61	0.48	0.487
Group * Block	1.99	68.5	0.97	0.377
Stimulus * Group	1.95	66.9	0.57	0.560
Stimulus * Group * Block	1.84	61	1.28	0.278
Recall				
Block	1	63.1	48.97	<.001 ***
Stimulus	1	64.1	0.13	0.716
Group	1.98	69.8	4.98	**0.007 ****
Stimulus * Block	1	70.7	3.28	0.070
Group * Block	1.9	63.1	3.04	0.050
Stimulus * Group	1.89	64.1	0.57	0.554
Stimulus * Group * Block	2	70.7	0.86	0.423

Block	1	71.4	32.73	<.001 ***
Stimulus	1	62.1	1.17	0.280
Group	1.98	70.3	0.09	0.916
Stimulus * Block	1	66.8	1.07	0.302
Group * Block	1.99	71.4	1.52	0.218
Stimulus * Group	1.85	62.1	0.13	0.860
Stimulus * Group * Block	1.93	66.8	0.35	0.699
Recall				
Block	1	61.4	57.08	<.001 ***
Stimulus	1	70.3	2.70	0.100
Group	1.99	70.6	0.04	0.960
Stimulus * Block	1	68.7	2.50	0.114
Group * Block	1.87	61.4	1.23	0.291
Stimulus * Group	1.99	70.3	0.38	0.682
Stimulus * Group * Block	1.97	68.7	0.52	0.594

First recall trials pupillometry and skin conductance responses

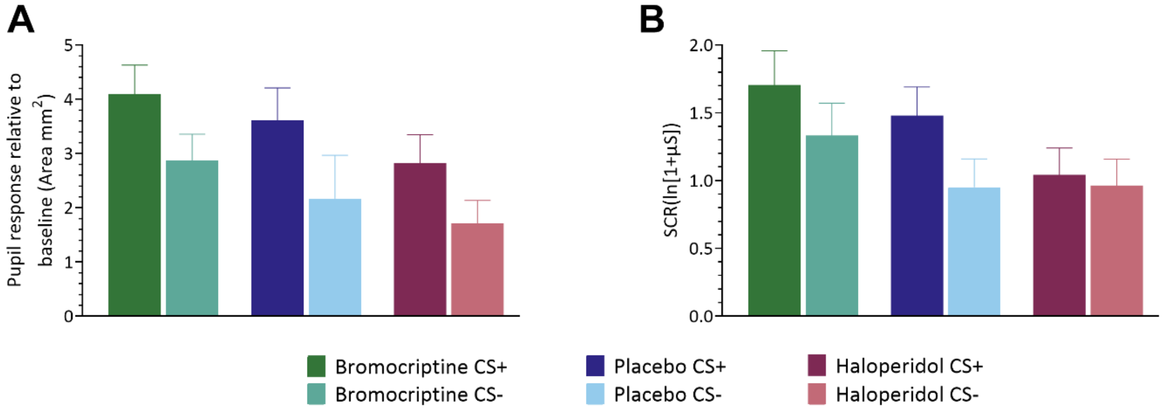

Figure S4 - (A) *Pupil responses relative to baseline for first recall trial in the* bromocriptine (n=24), placebo (n=25) and haloperidol (n=25) groups*.* Bars represent means, *error bars indicate S.E.M. (B) Skin conductance responses (SCRs) for first recall trial in the groups receiving* bromocriptine (n=25), placebo (n=25) and haloperidol (n=24). Bars represent means, *error bars indicate S.E.M. Separate non-parametric ANOVA-type statistics were conducted for pupil and SCR data, with medication subgroup as a between-subjects factor and stimulus type (CS+ vs. CS–) as a within-subjects factor. Post-hoc least square means comparisons were performed using Dunnett's adjustment to compare each drug group to its respective placebo. A significant main effect of stimulus was found for both pupil responses ($F_{(1)}$=8.23, p=0.004) and SCRs ($F_{(1)}$=8.66, p=0.003), indicating spontaneous recovery. No significant main effects of drug group were found (Pupil: p = 0.054; SCR: p=0.243), and no significant stimulus × group interactions were observed (Pupil: p=0.857; SCR: p=0.255).*

We assessed the first trial in recall across the bromocriptine, placebo, and haloperidol groups using both pupil dilation and SCR (*Figure S4–A and B; Table S11*). Both measures showed a significant difference between CS+ and CS- trials (Pupil: $F_{(1)}$=8.23, p=0.004; SCR: $F_{(1)}$=8.66, p=0.003), indicating spontaneous recovery of the initial fear association. However, there were no significant differences between the drug groups for either measure (Pupil: $F_{(1.93)}$=2.96, p=0.054; SCR: $F_{(1.99)}$=1.42, p=0.243). For the interaction between stimuli and drug group, neither measure showed a significant effect (Pupil: $F_{(1.77)}$=0.13, p=0.857; SCR: $F_{(1.97)}$=1.37, p=0.255; *Table S11)*.

Table S11 - Pupillometry and SCR statistics in the groups receiving bromocriptine, placebo and haloperidol. Results of the non-parametric ATS for repeated measures on the first trial of recall. (*p < 0.01; ** p < 0.05; *** p < 0.001)

Factor	Num DF	Den DF	F	Pr>F(infty)
**Pupillometry**				
Stimulus	1	54.6	8.23	0.004**
Group	1.93	62.1	2.96	0.054
Stimulus * Group	1.77	54.6	0.13	0.857
SCR				
Stimulus	1	68.5	8.66	0.003**
Group	1.99	69.8	1.42	0.243
Stimulus * Group	1.97	68.5	1.37	0.255

Exploratory analyses: Methods

We conducted exploratory analyses to complement our primary findings. First, to examine potential dose-dependent effects, we tested whether individual serum drug concentrations were associated with differential conditioned responses (CS+ minus CS−) during extinction and recall, using Spearman correlations separately for each drug and phase. Second, to assess the influence of baseline psychological states, we correlated DASS-21-G subscale scores (depression, anxiety, stress) with differential SCRs during acquisition for all participants and during extinction in placebo-treated participants. To correct for multiple comparisons across correlation test, we applied a false discovery rate (FDR) correction using the Benjamini–Hochberg procedure. Third, in line with previous findings (e.g., Gerlicher et al., 2019)[4], we tested whether the effect of each drug on recall-phase SCRs differed between participants with successful vs. unsuccessful extinction. Participants were classified as good extinguishers if their differential SCR during late extinction was ≤ 0. For each drug, a mixed-effects model was fitted with drug, extinction success (good vs. poor), and their interaction as fixed effects.

Exploratory analyses: Results

Pupil size variation at baseline

Table S12 - Pupil size variation (in mm^2) 2s prior CSs onset during the first 8 trials in both group A receiving levodopa, placebo or tiapride and group B receiving bromocriptine, placebo or haloperidol. Results of the non-parametric ATS for repeated measures between all phases inside each group. All *p*-values are adjusted using the Tukey–Kramer method for multiple comparisons. (* p<0.05; ** p<0.01; *** p<0.001)

Factor	Num DF	Den DF	F	Pr>F(infty)
		**Levodopa**		
Phase	2.99	728	4.38	**0.004 ****
		**Placebo A**		
Phase	3	738	1.08	0.357
		**Tiapride**		
Phase	3	682	22.94	**<.001*****
		**Bromocriptine**		
Phase	3	754	3.03	**0.028***
		**Placebo B**		
Phase	3	789	0.67	0.571
		**Haloperidol**		
Phase	3	794	5.64	**<.001*****

Drug concentration and conditioned response correlations

To explore potential dose-dependent effects of the administered drugs, we conducted exploratory Spearman correlation analyses between individual serum drug concentrations and the differential conditioned responses (CS+ minus CS−), measured via SCR and pupil size. Analyses were performed separately for each drug, across early and late blocks of both extinction and recall phases.

For levodopa and tiapride, Day 2 concentrations were used. For haloperidol, due to its extended half-life, we examined correlations using both Day 2 and Day 3 serum levels. Drug serum concentrations showed substantial interindividual variability, but no correlation reached statistical significance after correction for multiple comparisons. The only association approaching significance was a moderate negative correlation between tiapride concentration and SCR during early recall (r=–0.45, uncorrected p=0.035; *Table S13*), indicating that higher levels may be associated with attenuated SCRs. However, this effect did not survive correction and was not observed in other blocks or replicated in pupil responses. Overall, these findings do not support correlations between serum drug levels and conditioned response expression across the drugs and concentration ranges examined.

Table S13 - Spearman correlations between drug serum concentrations and differential conditioned responses (Skin Conductance Responses (SCRs) and pupillometry) during fear extinction and recall. Correlations are based on serum concentrations measured after the extinction phase (Day 2) and, where indicated, after the recall phase (Day 3). Statistical analyses were performed separately for each drug, phase, and block (early vs. late). Reported values include Spearman's correlation coefficient (r) and FDR-corrected p-values (p-FDR). (* p<0.05)

Based on drug concentration measured after extinction phase									
SCR					Pupillometry				
Extinction					Extinction				
Drug	Block	Spearman r	p-value	p-FDR	Drug	Block	Spearman r	p-value	p-FDR
Levodopa	Early	0.233	0.274	0.768	Levodopa	Early	-0.062	0.773	0.773
	Late	-0.014	0.947	0.947		Late	0.091	0.687	0.773
Tiapride	Early	0.138	0.540	0.768	Tiapride	Early	-0.168	0.466	0.743
	Late	0.106	0.637	0.811		Late	0.299	0.187	0.669
Haloperidol	Early	-0.138	0.510	0.768	Haloperidol	Early	-0.362	0.076	0.669
	Late	-0.020	0.924	0.947		Late	-0.164	0.434	0.743
Recall					Recall				
Drug	Block	Spearman r	p-value	p-FDR	Drug	Block	Spearman r	p-value	p-FDR
Levodopa	Early	-0.152	0.478	0. 768	Levodopa	Early	0.272	0.233	0.669
	Late	0.137	0.522	0.768		Late	0.145	0.531	0.743
Tiapride	Early	-0.453	0.035*	0.482	Tiapride	Early	-0.071	0.755	0.773
	Late	0.135	0.549	0.768		Late	0.115	0.610	0.773
Haloperidol	Early	-0.340	0.097	0.556	Haloperidol	Early	-0.147	0.483	0.743
	Late	-0.217	0.297	0.556		Late	-0.223	0.287	0.669
**Based on drug concentration measured after recall phase**									
SCR					Pupillometry				
Recall					Recall				
Drug	Block	Spearman r	p-value	p-FDR	Drug	Block	Spearman r	p-value	p-FDR
Haloperidol	Early	-0.036	0.867	0.947	Haloperidol	Early	-0.285	0.168	0.669
	Late	-0.320	0.119	0.556		Late	-0.224	0.282	0.669

DASS-21-G scores and conditioned response correlation

To explore whether individual differences in affective states were associated with conditioned responding, we conducted exploratory Spearman correlation analyses between self-reported DASS-21-G subscale scores (depression, anxiety, stress) and physiological measures of fear acquisition and extinction learning. Skin conductance response (SCR) and pupil size data were used to compute differential responding (CS+ minus CS−) for each participant. To avoid pharmacological confounds, we restricted extinction correlation analyses to placebo groups' participants. Acquisition correlation analyses included all participants. To quantify response magnitude and learning rate, we calculated the mean difference between the first three CS+ and the first three CS− trials (early phase) and between the last three CS+ and last three CS− trials (late phase) within each experimental phase (acquisition and extinction). Analyses were conducted separately for early and late acquisition and extinction phases (*Table S14*). Correlation coefficients were small to

moderate (|r|≤0.19) and none reached statistical significance (all p>0.05). Overall, these findings suggest that baseline DASS-21-G scores were not reliably associated with differential physiological responses during fear acquisition or extinction.

Table S14 - Spearman correlations between baseline depression, anxiety, and stress levels (DASS-21-G) and differential conditioned responses (Skin Conductance Responses (SCRs) and pupillometry) during fear acquisition and extinction. Reported values include Spearman's correlation coefficient (r) and FDR-corrected p-values (p-FDR).

SCR					Pupillometry				
Acquisition (N=146)					Acquisition (Early N=142 / Late N=140)				
DASS	Block	Spearman r	p-value	p-FDR	DASS	Block	Spearman r	p-value	p-FDR
Depression	Early	–0.029	0.730	0.975	Depression	Early	0.157	0.062	0.581
	Late	0.063	0.387	0.975		Late	-0.056	0.508	0.609
Anxiety	Early	–0.072	0.453	0.975	Anxiety	Early	-0.068	0.424	0.581
	Late	–0.004	0.966	0.975		Late	-0.079	0.357	0.581
Stress	Early	–0.068	0.416	0.975	Stress	Early	0.095	0.260	0.581
	Late	0.067	0.421	0.975		Late	-0.084	0.324	0.581
Extinction (N=50)					Extinction (N=48)				
DASS	Block	Spearman r	p-value	p-FDR	DASS	Block	Spearman r	p-value	p-FDR
Depression	Early	0.004	0.975	0.975	Depression	Early	-0.169	0.252	0.581
	Late	–0.080	0.583	0.975		Late	0.115	0.436	0.581
Anxiety	Early	–0.018	0.903	0.975	Anxiety	Early	-0.190	0.196	0.581
	Late	–0.029	0.839	0.975		Late	0.010	0.945	0.945
Stress	Early	0.181	0.209	0.975	Stress	Early	-0.143	0.334	0.581
	Late	0.042	0.775	0.975		Late	0.036	0.806	0.879

Fear recall in good and poor extinguishers across drug conditions

As preregistered and based on prior work[3], we tested whether the effects of dopaminergic drugs on fear recall differed depending on extinction success. Extinction success was defined based on differential SCRs (CS+ minus CS−) during the late extinction phase[3]. Participants with values ≤ 0 were classified as good extinguishers, while those with values > 0 were considered poor extinguishers. For each drug–placebo pair (Levodopa, Tiapride, Bromocriptine, Haloperidol), we ran a separate linear mixed-effects model with fixed effects of drug, extinction success, and their interaction, and participant as a random effect. The dependent variable was the differential SCR (CS+ minus CS−) on the first recall trial.

In the levodopa model, the interaction between drug and extinction success was not significant (p=0.605), indicating that the effect of levodopa on fear recall did not depend on whether participants had successfully extinguished fear prior. Similar analysis was performed for Tiapride, Bromocriptine, Haloperidol and their respective placebo. None of these compounds showed a significant interaction with extinction success.

Haloperidol showed a significant main effect, increasing differential SCRs at recall compared to placebo (p=0.050), while Tiapride and Bromocriptine showed no reliable effects. When plotting the

data, a potential outlier was identified in the haloperidol group; removing this data point rendered the effect non-significant ($p>0.05$). In addition, Levodopa was associated with higher overall SCR levels at recall compared to placebo ($p=0.036$), independent of extinction success. This effect is in line with previous results from our paper.

Supplementary references

1. Brunner E, Domhof S, Langer F. Nonparametric Analysis of Longitudinal Data in Factorial Experiments, J. Wiley, New York, NY, 2002.

2. Noguchi K, Gel YR, Brunner E, Konietschke F. nparLD : An R Software Package for the Nonparametric Analysis of Longitudinal Data in Factorial Experiments . J Stat Softw. 2012;50(12). doi:10.18637/jss.v050.i12

3. Bathke AC, Schabenberger O, Tobias RD, Madden L V. Greenhouse-Geisser adjustment and the ANOVA-type statistic: Cousins or twins? Am Stat. 2009;63(3):239-246. doi:10.1198/tast.2009.08187

4. Gerlicher AMV, Tüscher O, Kalisch R. L-DOPA improves extinction memory retrieval after successful fear extinction. Psychopharmacology (Berl). 2019;236(12):3401-3412. doi:10.1007/s00213-019-05301-4
